# The History and Diversity of Rice Domestication as Resolved From 1464 Complete Plastid Genomes

**DOI:** 10.3389/fpls.2021.781793

**Published:** 2021-11-18

**Authors:** Wenchuang He, Caijin Chen, Kunli Xiang, Jie Wang, Ping Zheng, Luke R. Tembrock, Deming Jin, Zhiqiang Wu

**Affiliations:** ^1^Shenzhen Branch, Guangdong Laboratory of Lingnan Modern Agriculture, Genome Analysis Laboratory of the Ministry of Agriculture and Rural Affairs, Agricultural Genomics Institute at Shenzhen, Chinese Academy of Agricultural Sciences, Shenzhen, China; ^2^MOA Key Laboratory of Crop Ecophysiology and Farming System in the Middle Reaches of the Yangtze River, College of Plant Science and Technology, Huazhong Agricultural University, Wuhan, China; ^3^Institute of Biological and Environmental Sciences, University of Aberdeen, Aberdeen, United Kingdom; ^4^School of Landscape and Architecture, Zhejiang A&F University, Hangzhou, China; ^5^Department of Horticulture, Washington State University, Pullman, WA, United States; ^6^Department of Agricultural Biology, Colorado State University, Fort Collins, CO, United States

**Keywords:** plant domestication, crop wild relatives, conservation genetics, phylogeography, germplasm resources

## Abstract

The plastid is an essential organelle in autotrophic plant cells, descending from free-living cyanobacteria and acquired by early eukaryotic cells through endosymbiosis roughly one billion years ago. It contained a streamlined genome (plastome) that is uniparentally inherited and non-recombinant, which makes it an ideal tool for resolving the origin and diversity of plant species and populations. In the present study, a large dataset was amassed by *de novo* assembling plastomes from 295 common wild rice (*Oryza rufipogon* Griff.) and 1135 Asian cultivated rice (*Oryza sativa* L.) accessions, supplemented with 34 plastomes from other *Oryza* species. From this dataset, the phylogenetic relationships and biogeographic history of *O. rufipogon* and *O. sativa* were reconstructed. Our results revealed two major maternal lineages across the two species, which further diverged into nine well supported genetic clusters. Among them, the Or-wj-I/II/III and Or-wi-I/II genetic clusters were shared with cultivated (percentage for each cluster ranging 54.9%∼99.3%) and wild rice accessions. Molecular dating, phylogeographic analyses and reconstruction of population historical dynamics indicated an earlier origin of the Or-wj-I/II genetic clusters from East Asian with at least two population expansions, and later origins of other genetic clusters from multiple regions with one or more population expansions. These results supported a single origin of *japonica* rice (mainly in Or-wj-I/II) and multiple origins of *indica* rice (in all five clusters) for the history of rice domestication. The massive plastomic data set presented here provides an important resource for understanding the history and evolution of rice domestication as well as a genomic resources for use in future breeding and conservation efforts.

## Introduction

The domestication and improvement of wild plant species forms the basis of our current global food system (Hancock, 2004). Among plant lineages that have been domesticated to feed humans directly, Asian cultivated rice (*Oryza sativa* L.) is one of the most important given that it provides roughly 20% of the calories consumed by humans worldwide (Smith, 1998). Like other core staple food crops, documenting and analyzing wild and cultivated genetic diversity is the basis for increasing yield and the development of insect/drought/disease resistant varieties (Brown, 1987; Valliyodan et al., 2017; Wang H. et al., 2018; Vikas et al., 2020). Currently, the genetic diversity (especially in the wild) of the crop species upon which humans depend for survival are profoundly threatened from forces such as habitat loss, climate change, regional conflict, human population growth, and anthropogenic vectoring of pathogens (Wilkes and Williams, 1983; Smith et al., 2006; Ford-Lloyd et al., 2011; Hoban et al., 2020). Thus, it is increasingly necessary to improve our understanding of rice domestication and genetic diversity.

With the advent of high-throughput DNA sequencing technology many genetic studies of crop origins have focused on allelic variation from whole nuclear genomes (Rojas-Barrera et al., 2019; Razifard et al., 2020) or domestication genes (Scott et al., 2019; Liu et al., 2020; Lu et al., 2020) with limited use of whole plastid genomes for inferring crop origins. However organellar genomes (in particular plastids) possess several advantageous qualities in the inference of crop origin and diversity including high copy number, which is useful in degraded archeological finds (Schlumbaum et al., 2008; Wagner et al., 2018), uniparental inheritance resulting in more recent inferences for coalescence times (Palumbi et al., 2001), lack of recombination reducing problems associated with introgression and incomplete lineage sorting (Hu et al., 2016), and a variable mutation rate which is useful for inferences at multiple taxonomic levels (Pollmann et al., 2005). There are numerous well-known examples using organellar DNA in studies of origin, dispersal, and diversity, like the resolution of our own human history using mitochondrial DNA (Ballinger et al., 1992; Forster et al., 2001; Tanaka et al., 2004) and the use of plastid DNA as a standard phylogenetic marker in the Angiosperm Phylogeny Group (APG) classification of flowering plant orders and families (Byng et al., 2016). Therefore, pangenomic approaches to plastomic DNA analyses are expected to provide useful insights into the origin, and diversity of numerous domesticated crop species.

As a staple food crop which feeds over 3.5 billion people on the planet, domestication of cultivated rice ranks as one of the most important developments in the history of humankind. Accordingly, the origins and demographic history of Asian cultivated rice (*O. sativa*) have been frequently studied using a wide range of analytical approaches mainly from the fields of genetics and archeology. Yet debates regarding the exact origin and timing of domestication persist (Londo et al., 2006; Caicedo et al., 2007; Fuller et al., 2009; Molina et al., 2011; Huang et al., 2012; Civan et al., 2015; Choi et al., 2017), especially regarding the number and source of origins with some researchers suggesting a single origin from one population while others proposed multiple distinct origins from different populations (Sang and Ge, 2007; Gross and Zhao, 2014; Civan et al., 2019). The single-origin hypotheses primarily focus on domestication genes (such as the non-shattering gene *sh4*) proposing that a desirable set of alleles occurred once, was selected for and that subsequent introgression with wild gene pools (while maintaining strong selection for the domestication genes) accounts for the current high diversity found in domesticated rice (Li C. B. et al., 2006; Sweeney et al., 2006; Vaughan et al., 2008). In contrast, the multi-origin hypotheses posit that distinct lineages of wild rice (*Oryza rufipogon*) in multiple locations were the source of different domesticated lineages (e.g., *indica* and *japonica*) and that the multi-origin sourcing accounts for most of the current diversity in domesticated rice rather than amalgamative introgression (Gross and Zhao, 2014).

In the present study, we constructed a large plastome data set containing 1445 complete *de novo* assembled genomes from wild and domesticated accessions in order to address the main outstanding questions in rice domestication: (1) Is the current diversity of domesticated rice derived from a single or multiple independent lineages? and (2) whether a common geographic origin or multiple geographic origins existed for the lineages? We employed numerous analytical approaches from population genetics and phylogeography to test these hypotheses. The results from these analyses were interpreted in light of previous work on rice history such that an updated model based on matrilineal diversity could be presented for this essential food crop. Our results from this large dataset of complete plastid genomes for wild and domesticated rice resolves distinct patterns in the evolution and domestication of different rice lineages. As with similar studies on the history and diversity of crop species the results herein will be directly applicable to crop improvement as well as *in situ* and *ex situ* conservation efforts.

## Materials and Methods

### Raw Dataset

Whole genome sequencing (WGS) data for *O. rufipogon* and *O. sativa* accessions were obtained from the EMBL database uploaded from previous studies (Huang et al., 2012; Wang W. S. et al., 2018). A total of 1445 samples, including 1135 *O. sativa* cultivars, 295 *O. rufipogon* accessions and 15 accessions from other wild rice species, were selected and collected from multiple regions in Asia, Europe, Africa, America, and Oceania (Supplementary Table 1 and Supplementary Figure 1). The samples were first selected randomly from the original datasets and then adjusted to ensure that they were distributed evenly according to geographic distribution. The accessions of *O. sativa* were classified into five categories including *indica*, *japonica* (including tropical *japonica*, typical *japonica*, and temperate *japonica*), *aus*, *aromatic*, and intermediate based on the identification included in the uploaded information (Wang W. S. et al., 2018). Nineteen published plastomes of *Oryza* species were downloaded from the NCBI database (Supplementary Table 2).

### The *de novo* Assembly and Annotation of Complete Plastid Genomes

The raw sequencing data were processed using FastQC v0.11.5 and NGSQCToolkit v2.3 software to control sequence quality, and were then filtered using BWA and SAMtools (Li and Durbin, 2010) software to extract plastid-origin reads that were properly paired to the reference plastid genomes from *Oryza* (Supplementary Table 3). The *de novo* assembly of complete plastid genomes were conducted using SPAdes (Bankevich et al., 2012). Several strategies were applied in the *de novo* assembly pipeline: (1) The problem of excessive variation introduced by using highly variable reference sets was reduced by using a narrower set of reference plastomes only from *Oryza* and (2) the F 12 option in SAMtools was used to bait target reads of plastomes from the WGS dataset. The obtained plastid contigs were further visualized and scaffolded to complete circular genomes in Bandage (Wick et al., 2015). The read coverage depth of each contig was used to identify and remove the mis-assembled contigs derived from nuclear or mitochondrial genomes, which were usually lower than one-fifth the depth of plastid contigs. Other optimized options were also applied as reported in our previous study (He et al., 2020).

Summary statistics were calculated to evaluate the quality of the assembled plastid genomes by using QUAST (Gurevich et al., 2013). Gene annotation was performed using Geseq (Tillich et al., 2017) and manually modified if necessary. Gene function annotation of nucleotide variations were performed with ANNOVAR software (Wang et al., 2010).

### Haplotype and Genetic Diversity Analyses

Population structure of all rice accessions were inferred based on plastomic single nucleotide variants (SNVs) by using Admixture software (Alexander et al., 2009), with 5 replications for different number of potential sub-populations (K) ranging from 1 to 25. The runs at each K showing the minimum cross-validation (CV) errors were used as optimal results to estimate the number of sub-populations. For the optimal K, the run with the lowest CV error was used to assign accessions with membership probability ≥0.65 to a sub-population (genetic cluster). Accessions with membership probability <0.65 were assigned to an unclassified group. Genealogical relationships of the identified haplotypes were inferred using a median joining method in Popart v1.7 (Leigh and Bryant, 2015) with annotations to the output figure completed in Adobe Illustrator software (Adobe Systems Incorporated, United States). Haplotype diversity, evolutionary distances based on the Tajima-Nei model, and population differentiation (*Fst*) were calculated for each group of haplotypes based on plastomic SNVs by using DnaSP v6.0 (Rozas et al., 2017) and MEGA7 (Kumar et al., 2016). The variational autoencoder plotting was calculated with popvae software (Battey et al., 2021). The principal coordinates analysis (PCoA) and multidimensional scaling (MDS) were conducted in TASSEL 5 (Bradbury et al., 2007). Nucleotide diversity (π) within and between different genetic clusters was calculated and tested using the pairwise differences method in Arlequin 3.5 (Excoffier and Lischer, 2010).

### Phylogenetic Analysis and Molecular Dating

To perform the phylogenetic analysis, complete sequences of plastid genomes were aligned in MAFFT (Katoh and Standley, 2013) and manually adjusted in MEGA7 (Kumar et al., 2016). Phylogenetic analyses were conducted using maximum likelihood methods in IQ-Tree (Nguyen et al., 2014), and the Bayes information criterion was used to determine the best-fit model for nucleotide substitution. Branch support was determined with ultrafast bootstrap of 2000 replicates. The final trees were then plotted using the online tool in the Interactive Tree of Life.^1^

For molecular dating, a set of representative haplotypes from major clades in the phylogenetic tree (consistent with the genetic clusters) was used for analyses. The smaller data set was used in order to simplify analyses and avoid zero-length branches from including repeat or highly similar haplotypes (Hou et al., 2014). A relaxed lognormal molecular clock with Yule priors was chosen to estimate the divergence time of lineages as implemented in BEAST v1.8.4 (Drummond et al., 2012). Three calibration points, from previous studies^2^ were employed: the divergence between *O. rufipogon* and *Oryza punctata* [5.7 mya 95% highest posterior density (HPD): 2.6–8.9], the crown age of *O. rufipogon* (2.32 mya HPD: 2–2.93), and the divergence time between *O. rufipogon* and *Oryza glaberrima* and its wild relative *Oryza barthii* (estimated at c. 0.83 mya HPD: 0.51–1.11). Following the suggestion of Ho (2007), we assigned a normally distributed prior for the three calibrations, in which standard deviations of 2, 0.3, and 0.2 were used, respectively.

The Markov Chain Monte Carlo procedure was run for 100 million generations, with sampling every 10,000 generations. Tracer v.1.7.1^3^ was used to assess appropriate burn-in and the adequate effective sample size values (>200). A burn-in of 25% was applied, and the maximum clade credibility (MCC) tree with the mean ages and 95% HPD intervals on nodes was produced in TreeAnnotator v1.8.4 (part of the BEAST package) and visualized in FigTree v.1.4.2.^4^

### Biogeographic Analyses and Historical Population Dynamics

For the ancestral range reconstruction, seven geographical areas based on the regions of endemism of rice were defined (Takhtajan, 1986; Wu and Wu, 1996). R package BioGeoBEARS (Matzke, 2013) was used to compare biogeographical models and estimate ancestral ranges of the *Oryza* populations using the MCMC tree from BEAST. Three biogeographical models were compared using a ML framework: Dispersal-Extinction-Cladogenesis (DEC) model (Ree and Smith, 2008), dispersal-vicariance analysis (DIVA) (Ronquist, 1997), and BayArea (Landis et al., 2013), with each model run with and without the + J parameter. The fit for the different models was assessed using AIC and AICc scores. The maximum range size was set to four, as no extant species occurs in more than four biogeographical regions. To test the robustness of the ancestral range reconstruction, we also used statistical dispersal-vicariance analysis (S-DIVA) (Yu et al., 2010) and DEC (Ree and Smith, 2008) in RASP v3.2 (Yu et al., 2015). MaxEnt species distribution modeling was used to predict past and current climatically suitable areas for different genetic clusters of wild rice (Phillips et al., 2017). Nineteen bioclimatic variables with the 2.5 arc-minute spatial resolution were downloaded from WorldClim 1.4^5^ (Hijmans et al., 2005). The neutrality test and mismatch distribution for plastomic variations of different groups were conducted using DnaSP v6.0 (Rozas et al., 2017). The reconstruction of population demography was estimated with stairway plot based on folded site frequency spectrum (SFS) as described in Liu and Fu (2015). The assumed mutation rate (μ) of plastomes in *Oryza* species was defined as 1.5 × 10^–10^ mutations per site per generation which was calculated in BEAST v1.8.4 (Drummond et al., 2012).

## Results

### *De novo* Assembly of Rice Plastomes Based on Whole Genome Sequencing Data

Based on the WGS data derived from the EMBL database (Huang et al., 2012; Wang W. S. et al., 2018), *de novo* assembly of 1445 plastid genomes (also referred to as plastomes) was completed from 1135 *O. sativa* and 295 *O. rufipogon* accessions and 15 in other rice species with AA genomes in *Oryza* (Table 1 and Supplementary Table 1). A total length of 7.0–224.8 Mb of reads was yielded per plastome when the original WGS data was mapped to the reference genome, with read coverage averaging 222.6×. The *de novo* assemblies of each plastome yielded from 3 to 67 contigs, with an average of 7.1, for each accession, which were further extended and connected according to *Kmer* depth and *De Bruijn* paths among them. Accordingly, a total of 1445 high-quality complete circular genomes were assembled for each of the 1445 rice accessions. The obtained plastomes had an average length of 134,527.3 bp, and varied from 134,518 to 134,695 bp, with an overall average GC content of 39.0%, varying from 39.0 to 39.5%. A typical quadripartite structure was resolved among all of the genomes and included two inverted repeats (IRs) with an average length of 20,802.7 bp and an average GC content of 44.3%, separated by a large single copy region (LSC) with an average length of 80,575.7 bp and an average GC content of 37.1%, and a small single copy region (LSC) with an average length of 12,346.2 bp and an average GC content of 33.3%. The highest GC content was observed in the IRs, followed by the LSC and SSC, suggesting that different functions (e.g., gene regulation; Smarda et al., 2014) and/or evolutionary biases exist between these regions.

**TABLE 1:** Characteristics of 1445 *de novo* assembled rice plastid genomes.

Assembly characteristics	Minimum	Maximum	Average
Cp mapped reads	84,623	2,700,911	361,293
Cp mapped bases (Mb)	7.0	224.8	29.9
Coverage (x)	52.1	1664.7	222.6
Assembled contigs	3	67	7.08
Assembled scaffolds	1	1	1
Size of completed cp genome (bp)	134,158	134,695	134,527.3
LSC size in bp	80,321	80,740	80,575.7
SSC size in bp	11,972	12,385	12,346.2
IR size in bp	20,738	20,809	20,802.7
Overall GC content (%)	39.0	39.5	39.0
GC content in LSC (%)	37.1	37.1	37.1
GC content in SSC (%)	33.3	33.5	33.3
GC content in IR (%)	44.3	44.4	44.3

### Variation Among Plastomes in *Oryza* Species With AA Genomes

To investigate differences in DNA sequences at the whole plastome level, comparative analyses and gene annotations were conducted for 1464 complete plastomes comprised of 1445 *Oryza* plastomes assembled in this study (Supplementary Table 1) and 19 plastomes downloaded from NCBI database (Supplementary Table 2) for 8 *Oryza* species (Supplementary Figure 2 and Supplementary Table 4). A total of 1896 nucleotide variations from 1642 loci were detected among the 1464 plastomes (Supplementary Figure 2). The types of variation across the entire dataset included 1287 SNVs, 431 small insertions or deletions with length of <50 bp (InDels), 74 small fragment substitutions with length of <50 bp (Sub.), 86 fragment length polymorphisms (FLPs), and 18 large structural variations (17 large insertions or deletions, 1 large substitution) >50 bp. Among them, 351 nucleotide variations were observed in coding regions and included 6 frameshift mutations, 5 non-frameshift indels, 10 block substitutions, 215 synonymous SNVs, 113 non-synonymous SNVs, and 2 stop-codon-gain loci. These mutations were located in the coding regions of 52 different genes, including 41 protein-coding genes, 8 transfer RNAs (tRNA), 2 ribosomal RNAs (rRNA), and 1 antisense gene.

### Maternal Phylogeny and Population Structure Reveal Distinct Genetic Clusters in Wild and Cultivated Rice

**FIGURE 1:**
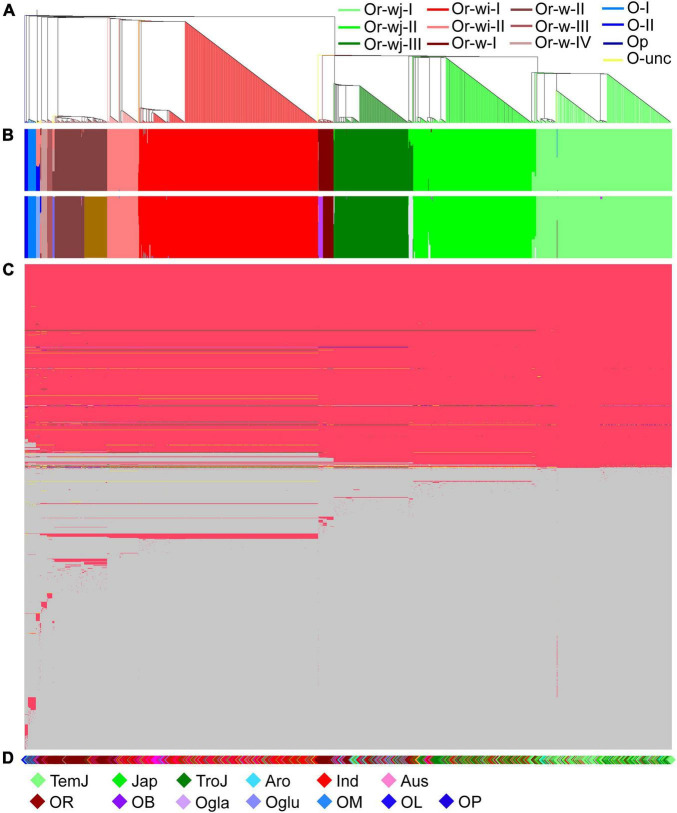
Plastomic relationships in all the wild and cultivated rice accessions. **(A)** Phylogenetic tree of 1464 plastomes in *Oryza* species with AA genomes and an outgroup species (*Oryza punctata* with BB genome). Different genetic clusters were denoted with different colors for the corresponding branches. **(B)** The two barplots indicate individual assignment posterior probabilities to a given group under the best partition of *K* = 12 and a secondary optimal partition of *K* = 17 as calculated in ADMIXTURE. **(C)** Plot illustrating all the plastome sequence differences across all accessions; A range of 2–20 different alleles, denoted with different colors, were detected at each variant locus in the 1464 accessions. In the diagram, the light blue indicates the ancestral alleles inferred from comparisons with the *Oryza punctata* plastome. **(D)** The corresponding species/cultivars were denoted for each accession: TemJ = Temperate *japonica*; Jap = typical *japonica*; TroJ = tropical *japonica*; Aro = *aromatic* rice; Ind = *indica* rice; Aus = *aus* rice; OR = *Oryza rufipogon*; OB = *O. barthii*; Ogla = *O. glaberrima*; Oglu = *O. glumipatula*; OM = *O. meridionalis*; OL = *O. longistaminata*; OP = *O. punctata*.

**FIGURE 2:**
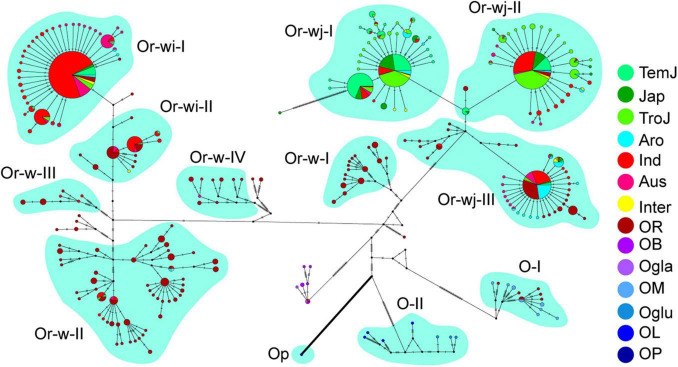
Haplotype network of plastomes in cultivated and wild rice species. Different cultivars/species were denoted in the haplotype network, and the haplotypes without a subgroup label belonged to the O-unc genetic cluster. The cultivars/species were designated for each accession as in Figure 1.

To investigate the plastomic relationships among all rice accessions, phylogenetic and population structure analyses were conducted, that consistently resulted in the resolution of multiple distinct genetic clusters with high support among both wild and cultivated accessions (Figure 1 and Supplementary Table 5). A total of 12 genetic clusters and 1 unclassified genetic cluster for all accessions were obtained from their phylogenetic relationship and the best partition of *K* = 12 as calculated in ADMIXTURE, although a secondary optimal partition of *K* = 17 was also observed (Figures 1A,B and Supplementary Figure 3). Two major maternal lineages were observed across all the *O. rufipogon* and *O. sativa* accessions, which could be further resolved into 4 *O. rufipogon*-wild-type genetic clusters (Or-w-I, Or-w-II, Or-w-III, and Or-w-IV), and 2 *O. rufipogon*-wild-and-cultivated-type clusters (Or-wj and Or-wi). The cultivated accessions were separated into Or-wj and Or-wi with multiple well-supported genetic clusters within each. Three *O. rufipogon*-wild-to-*japonica* (Or-wj) genetic clusters were resolved. The genetic clusters Or-wj-I and Or-wj-II mainly consisted of *japonica* cultivated varieties with some other cultivars, while Or-wj-III contained most of the *aromatic* rice cultivars that have often been considered as a related ecotype of *japonica* rice (Figure 1). Two *O. rufipogon*-wild-to-*indica* (Or-wi) genetic clusters were resolved and referred to as Or-wi-I and Or-wi-II. The Or-wi-I genetic cluster consisted of mainly *indica* rice cultivars and the aus rice ecotype, while the Or-wi-II genetic cluster contained *indica* cultivated rice and a high percentage (45.1%) of wild rice accessions (Figures 1, 2 and Supplementary Table 5). Almost all the wild rice accessions fell into one of four genetic clusters Or-w-I, Or-w-II, Or-w-III, or Or-w-IV, among which the Or-w-I was most closely related to the Or-wj genetic clusters and the Or-w-II was closest to the Or-wi genetic clusters (Figure 1A). Accessions from the other AA genome species (*O. barthii*, *O. glaberrima*, *Oryza glumipatula*, *Oryza meridionalis*, and *Oryza longistaminata*) resolved in genetic clusters *Oryza*-I (O-I), *Oryza*-II (O-II), and *Oryza*-unclassified (O-unc) with the BB genome species *O. punctata* (Op), used as the outgroup (Figure 1). The vast majority of cultivated rice accessions (1114/1137) were clustered in either the Or-wj or Or-wi groups. Notably, 23 domesticated accessions clustered into various wild genetic clusters indicating a more distant maternal relationship between them (Figures 1, 2).

### Nucleotide Divergence of Plastomes Within and Between Different Genetic Clusters

**FIGURE 3:**
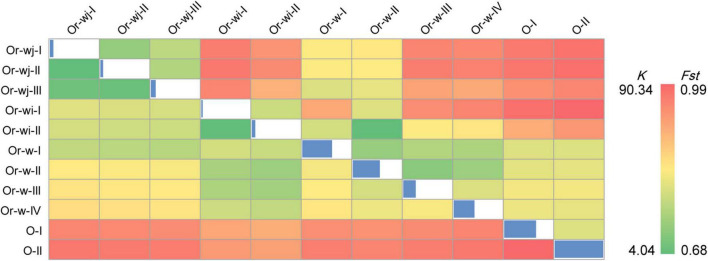
Genetic distances and population differentiation within and between different genetic clusters. Below diagonal: Pairwise genetic distances (*K*); diagonal: the nucleotide diversity (π) for each group; above diagonal: The population differentiation coefficient (*Fst*) between groups.

To further assess the nucleotide divergence of different clusters, a haplotype network of plastomes was constructed for all the accessions of wild and cultivated rice species from this study (Figure 2). In total 266 haplotypes were obtained from 1464 accessions which were resolved into 12 well supported genetic clusters and 1 mixed genetic cluster, which were classified as described above. Haplotype diversity in this study exhibited a similar pattern to other studies in grain crops where a broader diversity is found in wild haplotypes than in cultivated haplotypes. That is, clusters containing wild accessions exhibited a greater number of differences between haplotypes within the cluster, whereas haplotypes in the cultivated genetic clusters were separated only by a single or few steps (Figure 2). The wild rice (*O. rufipogon*) genetic clusters (Or-w groups) showed higher haplotype diversity (as a function of accessions per haplotype and multi-step branching within each genetic cluster) with 81 haplotypes in total and 1–11 accessions per haplotype. While cultivated rice genetic clusters (Or-wi and Or-wj groups) had 149 haplotypes in total with 1–301 accessions per haplotype. The nine most common haplotypes (>21 accessions) contained 77.3% of all the accessions in the cultivated rice genetic clusters. The cultivated clusters showed very low nucleotide diversity (π) with a range from 0.00047 to 0.0015 which is lower than wild genetic clusters ranging from 0.0035 to 0.014 (Figure 3 and Supplementary Figure 4). Similarly, the pairwise genetic distances within Or-wj (4.0–7.3) and Or-wi (4.2) were much lower than those of Or-w genetic clusters (31.4–41.9). This narrowing of genetic diversity is common among domesticated lineages wherein artificial selection and demographic bottlenecking can induce such patterns.

**FIGURE 4:**
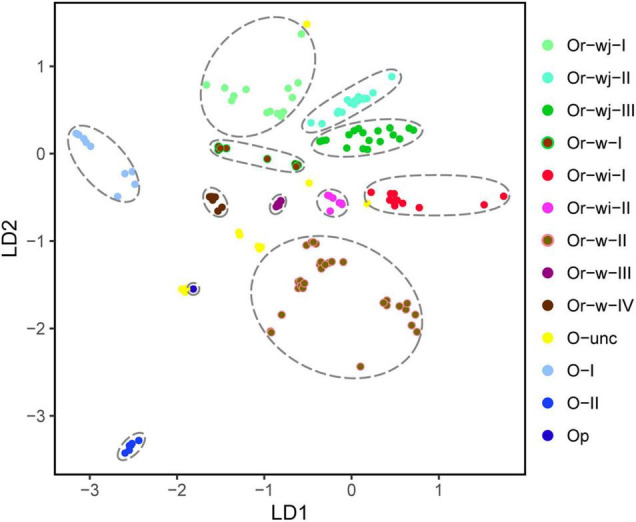
Variational autoencoder plotting for different genetic clusters in *Oryza* accessions.

Group and individual based calculations provided further evidence for a multiple origin with long-term artificial selection scenario for the domestication of rice. On average, each Or-wj accession differed from an Or-wi accession by 31.6 SNVs with a range of 29.4–34.0, which is roughly 8-fold more than detected between accessions within Or-wj (4.0, 6.0, and 7.3) or Or-wi (4.2) genetic clusters (Figure 3). When using *F*_*st*_ (calculated from SNVs) to assess divergence between different clusters, the Or-wj genetic clusters all showed relatively large population differentiation to Or-wi genetic clusters with a range of *F*_*st*_ from 0.946 to 0.979 at a significance level of *p* < 0.01. These values are larger than the range of *F*_*st*_ between either the Or-wj genetic clusters and Or-w-I (*F*_*st*_ = 0.859–0.914; *p* < 0.01) or between Or-wi genetic clusters and Or-w-II (*F*_*st*_ = 0.675, 0.863; *p* < 0.01). Additionally, the Or-wj genetic clusters showed the least genetic distance from Or-w-I (*K* = 24.3–26.3) while Or-wi genetic clusters were the least distant from Or-w-II (*K* = 18.2, 20.7). The variational autoencoder estimation (VAE), principal component analysis (PCA), and MDS plot were conducted to further investigate the genetic relationships of all the accessions based on plastomic SNVs. From all multidimensional individual based analyses, the accessions in Or-wj and Or-wi genetic clusters were grouped together in a similar manner to the other analyses and resolved closest to Or-w-I and Or-w-II, respectively (Figure 4 and Supplementary Figure 5). All of these results consistently resolve a pattern wherein the two genetic clusters containing mainly cultivated accessions were selected from two distinct wild lineages, thus supporting a multiple maternal genetic origin scenario for the history of rice domestication.

### Molecular Dating, Phylogeography, and Historical Demography Reconstructions for the Origin and Timing of Rice Domestication

**FIGURE 5:**
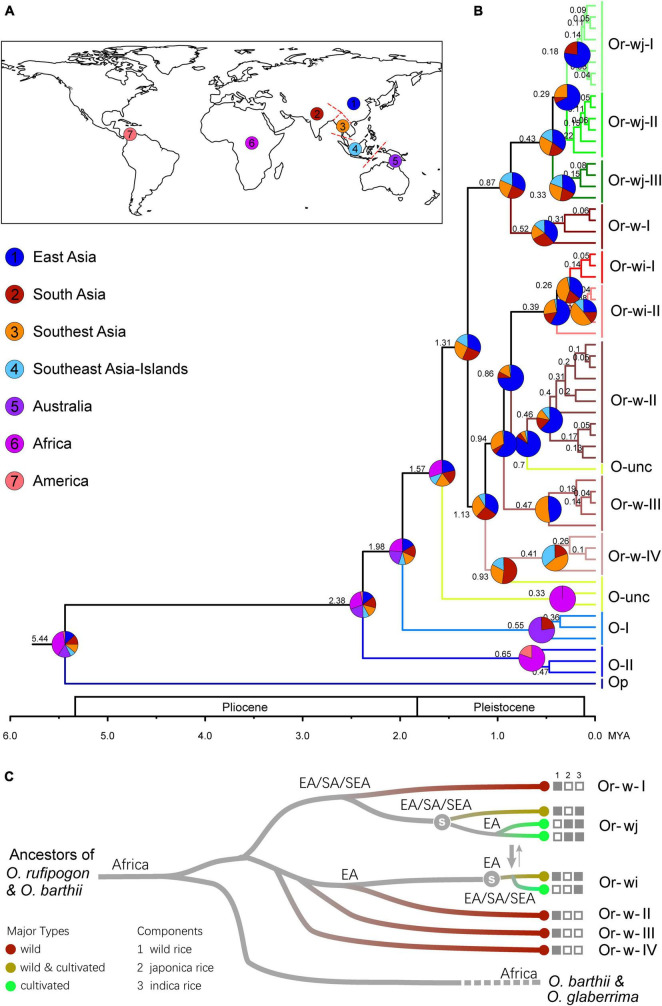
The origin and divergence patterns of cultivated rice and wild progenitors. **(A)** The delimitations of geographic origins used for the biogeographic reconstruction of wild and cultivated rice lineages. **(B)** Inferred phylogeographic patterns for the origin and dispersal of wild and cultivated rice genetic clusters. Pie charts represent probability of origin for a given clade. **(C)** Schematic of the origin and the inferred maternal lineages of cultivated rice and their wild relatives based on the results of the phylogeographic analyses. The white character “s” on the tree denotes when artificial selection may have started among each lineage.

To infer the historical dynamics of origin and evolution for wild and cultivated rice, a total of 61 representative plastid haplotypes from the cultivated and wild rice genetic clusters were employed in molecular dating and historical biogeographic reconstruction (Figure 5 and Supplementary Figures 6, 7). From molecular dating the clade of *O. rufipogon* and *O. sativa* diverged from a clade containing *O. barthii* and *O. glaberrima* (an African species of cultivated rice) approximately 1.6 mya. This indicated that the wild progenitors of the two cultivated rice species may have originated from a common ancestral lineage. The *O. rufipogon* and *O. sativa* clade diverged into two major lineages 1.31 mya and subsequently split up into multiple genetic clusters before 0.86 mya. The Or-wj genetic clusters have a stem age of 0.87 mya and further diverged into two mainly cultivated genetic clusters Or-wj-I (99.3% were cultivated accessions) and Or-wj-II (95.6%) and a wild-cultivated cluster Or-wj-III (61.7%) 0.43 mya (Supplementary Table 5 and Figures 5B,C). The Or-wi genetic clusters have a stem age of 0.86 mya and further diverged into a mainly cultivated genetic cluster Or-w-I (97.3% were cultivated accessions) and a wild-cultivated cluster Or-w-II (54.9%) 0.26 mya (Supplementary Table 5 and Figures 5B,C). From the phylogeographic analyses the best supported scenario for Or-wj groups is an independent single origin of Or-wj-I/II from East Asia and multiple origins of Or-wj-III (Supplementary Table 6 and Figure 5). For the Or-wi genetic clusters the best supported origin was from East Asia with dispersal and secondary diversification in Southeast and South Asia (Figure 5 and Supplementary Figures 7, 8). These results revealed a pre-domestication landscape of the divergency of different maternal lineages in wild and cultivated rice.

**FIGURE 6:**
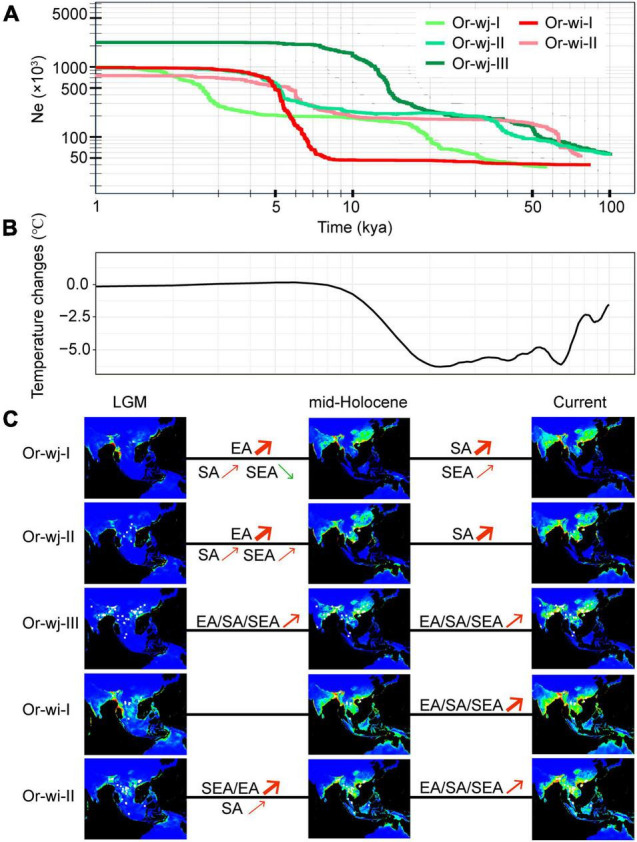
Historical dynamics of range and effective population size in different genetic clusters during the last 100,000 years. **(A)** Changes of the effective population sizes of the cultivated genetic clusters based on stairway plot estimations. **(B)** Change in Global Average Surface Temperature (GAST) for the last 100,000 years, compared with the average present temperature (0–5 kya), data were collected from Snyder (2016). **(C)** Potential distribution probability of occurrence for different genetic clusters of wild rice inferred by Maxent modeling. Warmer colors show areas with higher probabilities: red denotes the maximum value (1) and blue the minimum (0). White dots show the presence locations used for training. LGM, Last Glacial Maximum; mid-Holocene, middle Holocene. EA, East Asia; SA, South Asia; SEA, Southeast Asia. Red arrows indicate the expansion of geographic distribution for the population in a region while green arrows indicate the contraction of that population. These regions were defined the same as in Figure 5A.

From neutrality (Supplementary Table 7) and mismatch distribution tests (Supplementary Figure 9) all Or-wj and Or-wi genetic clusters were inferred to have undergone relatively recent population expansion which could be related to recent artificial selection associated with domestication compared to natural radiations among the wild progenitor lineages. Reconstruction of past effective population sizes and population historical distribution among the different groups suggests that population expansion occurred in different regions at different times between the genetic clusters (Figure 6). While analysis of all the Or-wj genetic clusters indicated a two-step population expansion pattern, the increases were not synchronous across genetic clusters with the first rapid increase in effective population occurring around 50 kya in Or-wj-II and Or-wj-III whereas in Or-wj-I this step occurs at about 15 kya. Similarly, the second rapid increases in effective population size between the Or-wj genetic clusters were asynchronous in all instances. Between the two Or-wi genetic clusters, inferred rapid expansion events were asynchronous in number, timing, and extent (Figure 6 and Supplementary Figure 8). Nearly all of the recently inferred expansion events appear to have occurred between 5.0 and 10.3 kya, except for Or-wj-I which was estimated to be more recent (2.0–3.0 kya). Modeling for climatically suitable areas of different genetic clusters suggested that all Or-wj and Or-wi clusters underwent geographic expansions both during periods of LGM to mid-Holocene and mid-Holocene to the present except for Or-wi-I (Figure 6C). The Or-wj-I/II were inferred to have a first dramatic expansion in East Asia and secondary expansion mainly in South Asia, while the other clusters appeared to expand in multiple regions in most periods. In total these results from different analytical approaches are consistent with the expectation of a multiple-origin scenario.

## Discussion

### Large Scale *de novo* Assemblies of Rice Plastid Genomes

Plastid has been a part of plant cells for roughly one billion years and remains the essential compartment for harvesting light energy in the cells of eukaryotic photosynthetic organisms. The plastid genome (plastome) possesses a number of special characteristics such as lack of recombination, conserved gene order and content, and differential rates of mutation (Supplementary Figure 2). These characteristics make plastomes ideally suited in the study of evolution and historical biogeography in plants (Gitzendanner et al., 2018), intergenomic transfer between organelles and nuclear genomes (Ma et al., 2020), plastid genetic engineering (South et al., 2019), and breeding and conservation of important crop germplasm (Tonti-Filippini et al., 2017; Gao et al., 2019). However, among the ∼35,000 cultivated species (Khoshbakht and Hammer, 2008), fewer than 80 complete plastid genomes are available in the NCBI database. Although the One Thousand Plants Transcriptome Project (1KP)^6^, as well as other recent efforts have contributed over 1,000 complete or nearly complete plastid genomes to global databases, most of these are from plants that are not of economic importance (Gitzendanner et al., 2018; Leebens-Mack et al., 2019; Li H. T. et al., 2019). Additionally, several large-scale datasets analyzing plastomic variations of wild and cultivated rice accessions were recently published, however these studies relied on a mapping-reads-to-a-reference method instead of *de novo* assembly and complete genome scaffolding (Civan et al., 2019; Moner et al., 2020). As such these analyses may have omitted regions of greater dissimilarity as these methods do not perform for longer genomic regions of this type. To date, there remains a lack of *de novo* assemblies for complete plastid genomes available in public databases that are sampled at the population level for important crop species.

In the present study, to conduct large scale *de novo* assemblies of well over 1000 plastid genomes, we developed an accurate and effective pipeline for auto-assembly of rice plastid genomes from high throughout WGS data. Several strategies were employed to obtain the optimal result of complete high-quality plastid genomes. The strategies included: (1) using only 102 plastomes in the *Oryza* genus as reference, instead of using a collection of dozens of distantly related species for reference which is the common practice; (2) applying the F 12 option when using SAMtools software to bait target reads of plastomes from the WGS dataset which resulted in improved baiting efficiency of plastomic reads from the WGS dataset and reduced non-target reads. Assembly parameters were also optimized according to the methods previously reported in He et al. (2020). Using this pipeline, we were able to assembly a total of 1445 completed plastomes of wild and cultivated rice accessions, with a high average depth of read coverage (222.6×). In order to confirm the accuracy of our assembly pipeline, two previously published plastomes for rice, cultivars Nipponbare (GU592207.1, AY522330.1) and TN1 (NC_031333.1) were selected to compare with the plastomes of the two accessions generated in this study (CX140 and CX162), respectively. Both genome sequences showed identical alignments with no nucleotide changes between them, further confirming the high reliability of the assembled plastome sequences generated by our pipeline. The results of this effort established a large set of 1445 complete plastomes including 1135 plastomes of Asian cultivated rice varieties and 310 plastomes of wild rice accessions. This dataset will be a very useful resource for future rice research on molecular diversity and phylogeny, plastid genetic engineering in rice, marker assisted breeding, and germplasm preservation efforts.

### Multiple Primary Origins With Ongoing Selection and Admixture Generate Secondary Origins Resulting in a High Diversity of Cultivated Rice Haplotypes

While much work has been dedicated to understanding the origin and evolution of domesticated rice the issue of multiple versus single origins is a topic of ongoing debate. Most recent studies have concluded that a multiple origin scenario is most likely however the number of domestication events wherein key grain domestication loci were acquired remains unresolved (Huang et al., 2012; Civan et al., 2015, 2019; Choi et al., 2017; Choi and Purugganan, 2018; Civan and Brown, 2018; Wang W. S. et al., 2018; Moner et al., 2020). The two major type of cultivated rice, *indica* and *japonica*, originated from different ancestral wild rice populations, with gene flows observed from *japonica* to *indica* as well as two relatively small ecotypes, *aus* and *aromatic* rice. The plastome data in this study provides more strong evidence for multiple origins of Asian cultivated rice and further resolution of the maternal relationships between its different subspecies/groups (Supplementary Figure 9). The cultivated rice and its wild relatives both derived from two major maternal lineages which could be traced back to a most recent common ancestor before 1.31 mya (Figure 5B). Extensive and directional introgressions were indicated between different cultivated groups according to polyphyletic cultivar designations to different plastid lineages, e.g., a mainly *japonica* (61.9%) type of genetic cluster Or-wj-II contained a large proportion of *indica* accessions (26.3%); more than half (58.2%) of *aromatic* rice accessions shared a common maternal lineage in the genetic cluster Or-wj-III, in which 31.6% accessions were *indica* or *aus* rice (Figures 1, 2 and Supplementary Table 5). Those results suggested abundant introgressions from *japonica* and *aromatic* rice to *indica* and *aus* rice cultivars. Furthermore, when comparing the different cultivated genetic clusters by using demographic and phylogeographic reconstruction the patterns appear to vary among the groups suggesting differences in geographic origin, intensity targets of selection, as well as timing of the initiation of these steps. It should be noted that the process of domestication by relaxing natural selection and increasing effective population sizes might have biased divergence time estimates. From the stairway plots (Figure 6) the inferred early diverging domesticated genetic clusters Or-wj-III and Or-wi-II had secondary rapid increases in effective population size starting around 15 and 7 kya, respectively. These times correspond roughly with dates inferred from other studies for when rice domestication could have reasonably started in China for *japonica* rice and in India for *indica* rice (Gross and Zhao, 2014). The older increases inferred in effective population size may have been the result of population responses to climatic changes related to past glacial events. Because the increases in effective population size are inferred to have occurred earlier in *japonica* genetic clusters, a scenario in which *Sh4* and other genes evolved once in East Asian *japonica* and were introgressed into *indica* lines is not unreasonable but also not the only explanation for this pattern. Evidence for introgression is present in this plastome dataset in the form of polyphyletic cultivar designations to different plastid lineages.

While plastid genomes are not generally considered to possess genes involved with the domestication syndrome the patterns found in plastomic data nonetheless provide invaluable data for reconstructing the domestication history of a crop species. For instance, the loss of nucleotide diversity is a common feature of grain domestication (Haudry et al., 2007; Gross and Olsen, 2010; Avni et al., 2017) and was evident in our plastid data as well. While nucleotide diversity was low in the domesticated groups compared to the wild groups, haplotype diversity was higher (*Hd*: Or-wj-I = 0.744, Or-wj-II = 0.887, Or-wj-III = 0.801; Supplementary Table 8). However, the high haplotype diversity in cultivated groups is mainly the result of many single step changes from one or two highly abundant haplotypes possibly reflecting the relaxation of natural selection and correlated increase in effective population sizes among domesticated lineages (Petit and Barbadilla, 2009; Liu et al., 2019). This contrasts with the wild genetic clusters wherein many multiple step branching events typify the diversity of haplotypes (Figure 4 and Supplementary Figure 10). Similar patterns between progenitor and derived domesticated lineages have been described for numerous cultivated and domesticated species (Bjornerfeldt et al., 2006; Miller and Gross, 2011; Tembrock et al., 2017; Alves-Pereira et al., 2018). Because plastids are not thought to be a target of selection during domestication and are mostly uniparentally inherited, they are considered superb markers to avoid the interruption of artificial intervention to the genomic mutation rate in resolving the geographic origins and timing of domestication in crop species. While these and similar characteristics do make plastid data well suited for resolving questions of crop origin such data are also not expected to be entirely neutral during domestication as plastids are important in numerous organismic processes such as environmental adaptation, growth rate, and cytonuclear incompatibility during reproduction (Allen, 2005; Greiner et al., 2011; Roux et al., 2016; Bogdanova, 2019; Flood et al., 2020). By combining plastome data with data from nuclear sequences, phenotype, and crop characteristics the degree to which plastid genes are involved in the process of domestication and improvement can be more effectively assessed.

### Cultivar Determination and Plastome Based Population Structure in Rice

Thousands (more than 40,000) of rice cultivars have been described since the beginning of documentation of rice cultivation (Li R. et al., 2019). Distinctive morphological traits and growth characteristics have been the primary means to recognize rice cultivars (Fujita et al., 1984; Li R. et al., 2019). More recently large genetic and genomic data sets have been employed to characterize rice diversity and understand the genetic underpinnings of desired traits (Kovi et al., 2011; Huang et al., 2013; Choi and Purugganan, 2018).

In our analysis of population structure using plastomic data most of the *japonica* (90.8%) and *aromatic* (86.6%) cultivars (determined *a prior* by original authors using cultivar traits) were present in the Or-wj genetic clusters. While the *indica* (67.0%) and *aus* (63.5%) cultivars (also determined *a priori* by original authors using cultivar traits) were mainly present in the Or-wi genetic clusters. From this it appears that *japonica* and *aromatic* cultivars are more closely associated with the Or-wj genetic clusters than the *indica* and *aus* cultivars are associated with the Or-wi genetic clusters. Such a pattern might be the result of biased maternal introgression from the Or-wj genetic clusters into *indicia* and *aus* cultivars. However, the inferred introgression of plastomes from the Or-wj genetic cluster into *indica* and *aus* varieties do not appear to have resulted in morphological changes that made them unrecognizable as *indicia* or *aus* cultivars. That said the influence of the Or-wj plastomes on the *indica* and *aus* traits might be minimal or they could be expressed in traits typically unrelated to cultivar recognition such as changes to photosynthetic rate.

The higher rate of inferred plastomic introgression from the Or-wj into the *indica* and *aus* cultivars raise several questions. For instance, is this pattern of asymmetrical introgression evidence of the transfer of the *sh4* and other domestication genes from gene pools containing the desired traits to gene pools without the desired traits as proposed in Choi and Purugganan (2018)? Has unidirectional cytoplasmic incompatibility resulted in higher rates of Or-wj plastomes in *indica* and *aus* cultivars? Do Or-wj plastomes provide desired traits not found in Or-wi plastomes to *indica* and *aus* cultivars? Obviously further work is needed to better understand how plastids are involved in the domestication process generally and in rice specifically. With datasets such as those presented here these questions are now possible to investigate more thoroughly. The outcome of such studies could be directly applicable in the development of new rice cultivars through plastomic marker assisted breeding and editing of key plastid genes. In addition, geographic locations with high plastomic haplotype diversity can be identified and prioritized for conservation of valuable wild rice germplasm such that they can be preserved for use in future rice breeding efforts.

## Data Availability Statement

The datasets presented in this study can be found in online repositories. The names of the repository/repositories and accession number(s) can be found below: https://www.ebi.ac.uk/ena, PRJEB43684 and https://ngdc.cncb.ac.cn/, PRJCA006880.

## Author Contributions

WH, ZW, DJ, and LT conceived and designed the study. WH, CC, KX, JW, and PZ collected the public data and performed the data analysis. WH, CC, and KX wrote the manuscript. ZW, LT, and DJ revised the manuscript. All authors have read and agreed to the published version of the manuscript and reviewed the manuscript.

## Conflict of Interest

The authors declare that the research was conducted in the absence of any commercial or financial relationships that could be construed as a potential conflict of interest.

## Publisher’s Note

All claims expressed in this article are solely those of the authors and do not necessarily represent those of their affiliated organizations, or those of the publisher, the editors and the reviewers. Any product that may be evaluated in this article, or claim that may be made by its manufacturer, is not guaranteed or endorsed by the publisher.

## Footnotes

1
https://itol.embl.de/

2
http://www.timetree.org/

3
http://beast.bio.ed.ac.uk/Tracer/

4
http://beast.bio.ed.ac.uk/FigTree

5
http://www.worldclim.org

6
https://db.cngb.org/onekp/

## References

[B1] Alexander D. H. Novembre J. Lange K. (2009). Fast model-based estimation of ancestry in unrelated individuals. *Genome Res.* 19 1655–1664. 10.1101/gr.094052.109 19648217 PMC2752134

[B2] Allen J. O. (2005). Effect of teosinte cytoplasmic genomes on maize phenotype. *Genetics* 169 863–880. 10.1534/genetics.104.027300 15731518 PMC1449101

[B3] Alves-Pereira A. Clement C. R. Picanco-Rodrigues D. Veasey E. A. Dequigiovanni G. Ramos S. L. F. (2018). Patterns of nuclear and chloroplast genetic diversity and structure of manioc along major Brazilian Amazonian rivers. *Ann. Bot.* 121 625–639. 10.1093/aob/mcx190 29309531 PMC5853005

[B4] Avni R. Nave M. Barad O. Baruch K. Twardziok S. O. Gundlach H. (2017). Wild emmer genome architecture and diversity elucidate wheat evolution and domestication. *Science* 357 93–97. 10.1126/science.aan0032 28684525

[B5] Ballinger S. W. Schurr T. G. Torroni A. Gan Y. Y. Hodge J. A. Hassan K. (1992). Southeast Asian mitochondrial DNA analysis reveals genetic continuity of ancient mongoloid migrations. *Genetics* 130 139–152. 1346259 10.1093/genetics/130.1.139 PMC1204787

[B6] Bankevich A. Nurk S. Antipov D. Gurevich A. A. Dvorkin M. Kulikov A. S. (2012). SPAdes: a new genome assembly algorithm and its applications to single-cell sequencing. *J. Comput. Biol.* 19 455–477. 22506599 10.1089/cmb.2012.0021 PMC3342519

[B7] Battey C. J. Coffing G. C. Kern A. D. (2021). Visualizing population structure with variational autoencoders. *G3 (Bethesda)* 11:jkaa036. 10.1093/g3journal/jkaa036 33561250 PMC8022710

[B8] Bjornerfeldt S. Webster M. T. Vila C. (2006). Relaxation of selective constraint on dog mitochondrial DNA following domestication. *Genome Res.* 16 990–994. 10.1101/gr.5117706 16809672 PMC1524871

[B9] Bogdanova V. S. (2019). Genetic and molecular genetic basis of nuclear-plastid incompatibilities. *Plants (Basel)* 9:23. 10.3390/plants9010023 31878042 PMC7020172

[B10] Bradbury P. J. Zhang Z. Kroon D. E. Casstevens T. M. Ramdoss Y. Buckler E. S. (2007). TASSEL: software for association mapping of complex traits in diverse samples. *Bioinformatics* 23 2633–2635. 17586829 10.1093/bioinformatics/btm308

[B11] Brown W. L. (1987). Global crop resources: gene banks and the world’s food. *Science* 236 617–618. 10.1126/science.236.4801.617 17740483

[B12] Byng J. W. Chase M. W. Christenhusz M. J. M. Fay M. F. Judd W. S. Mabberley D. J. (2016). An update of the angiosperm phylogeny group classification for the orders and families of flowering plants: APG IV. *Bot. J. Linnean Soc.* 181 1–20.

[B13] Caicedo A. L. Williamson S. H. Hernandez R. D. Boyko A. Fledel-Alon A. York T. L. (2007). Genome-wide patterns of nucleotide polymorphism in domesticated rice. *PLoS Genet.* 3:1745–1756. 10.1371/journal.pgen.0030163 17907810 PMC1994709

[B14] Choi J. Y. Platts A. E. Fuller D. Q. Hsing Y. I. Wing R. A. Purugganan M. D. (2017). The rice paradox: multiple origins but single domestication in asian rice. *Mol. Biol. Evol.* 34 969–979. 10.1093/molbev/msx049 28087768 PMC5400379

[B15] Choi J. Y. Purugganan M. D. (2018). Multiple origin but single domestication led to *Oryza sativa*. *G3 Gen. Genom. Genet.* 8 797–803. 10.1534/g3.117.300334 29301862 PMC5844301

[B16] Civan P. Ali S. Batista-Navarro R. Drosou K. Ihejieto C. Chakraborty D. (2019). Origin of the aromatic group of cultivated rice (*Oryza sativa* L.) traced to the Indian subcontinent. *Genome Biol. Evolu.* 11 832–843. 10.1093/gbe/evz039 PMC6427689 30793171

[B17] Civan P. Brown T. A. (2018). misconceptions regarding the role of introgression in the origin of *Oryza sativa* subsp. indica. *Front. Plant Sci.* 9:1750. 10.3389/fpls.2018.01750 30555497 PMC6282103

[B18] Civan P. Craig H. Cox C. J. Brown T. A. (2015). Three geographically separate domestications of Asian rice. *Nat. Plants* 1:15164. 10.1038/nplants.2015.164 27251535 PMC4900444

[B19] Drummond A. J. Suchard M. A. Xie D. Rambaut A. (2012). Bayesian phylogenetics with BEA Uti and the BEAST 1.7. *Mol. Biol. Evolu.* 29 1969–1973. 10.1093/molbev/mss075 PMC3408070 22367748

[B20] Excoffier L. Lischer H. E. L. (2010). Arlequin suite ver 3.5: a new series of programs to perform population genetics analyses under linux and windows. *Mol. Ecol. Res.* 10 564–567. 10.1111/j.1755-0998.2010.02847.x 21565059

[B21] Flood P. J. Theeuwen T. P. J. M. Schneeberger K. Keizer P. Kruijer W. Severing E. (2020). Reciprocal cybrids reveal how organellar genomes affect plant phenotypes. *Nat. Plants* 6 13–21. 10.1038/s41477-019-0575-9 31932677

[B22] Ford-Lloyd B. V. Schmidt M. Armstrong S. J. Barazani O. Engels J. Hadas R. (2011). Crop wild relatives-undervalued, underutilized and under threat? *Bioscience* 61 559–565.

[B23] Forster P. Torroni A. Renfrew C. Rohl A. (2001). Phylogenetic star contraction applied to Asian and papuan mtDNA evolution. *Mol. Biol. Evol.* 18 1864–1881. 10.1093/oxfordjournals.molbev.a003728 11557793

[B24] Fujita K. Coronel V. P. Yoshida S. (1984). Grain-filling characteristics of rice varities (*Oryza sativa* l.) differing in grain size under controlled environmental conditions. *Soil Sci. Plant Nut.* 30 445–454. 10.1080/00380768.1984.10434709

[B25] Fuller D. Q. Qin L. Zheng Y. Zhao Z. Chen X. Hosoya L. A. (2009). The domestication process and domestication rate in rice: spikelet bases from the lower yangtze. *Science* 323 1607–1610. 10.1126/science.1166605 19299619

[B26] Gao L. Z. Liu Y. L. Zhang D. Li W. Gao J. Liu Y. (2019). Evolution of oryza chloroplast genomes promoted adaptation to diverse ecological habitats. *Commun. Biol.* 2:278. 10.1038/s42003-019-0531-2 PMC6659635 31372517

[B27] Gitzendanner M. A. Soltis P. S. Wong G. K. S. Ruhfel B. R. Soltis D. E. (2018). Plastid phylogenomic analysis of green plants: a billion years of evolutionary history. *Am. J. Bot.* 105 291–301. 10.1002/ajb2.1048 29603143

[B28] Greiner S. Rauwolf U. Meurer J. Herrmann R. G. (2011). The role of plastids in plant speciation. *Mol. Ecol.* 20 671–691. 10.1111/j.1365-294X.2010.04984.x 21214654

[B29] Gross B. L. Olsen K. M. (2010). Genetic perspectives on crop domestication. *Trends Plant Sci.* 15 529–537. 10.1016/j.tplants.2010.05.008 20541451 PMC2939243

[B30] Gross B. L. Zhao Z. (2014). Archaeological and genetic insights into the origins of domesticated rice. *Proc. Natl. Acad. Sci. U.S.A.* 111 6190–6197. 10.1073/pnas.1308942110 24753573 PMC4035933

[B31] Gurevich A. Saveliev V. Vyahhi N. Tesler G. (2013). QUAST: quality assessment tool for genome assemblies. *Bioinformatics* 29 1072–1075. 10.1093/bioinformatics/btt086 23422339 PMC3624806

[B32] Hancock J. F. (2004). *Plant Evolution and the Origin of Crop Species.* Cambridge: CABI pubulishing.

[B33] Haudry A. Cenci A. Ravel C. Bataillon T. Brunel D. Poncet C. (2007). Grinding up wheat: a massive loss of nucleotide diversity since domestication. *Mol. Biol. Evol.* 24 1506–1517. 10.1093/molbev/msm077 17443011

[B34] He W. Chen C. Adedze Y. M. N. Dong X. Xi K. Sun Y. (2020). Multicentric origin and diversification of atp6-orf79-like structures reveal mitochondrial gene flows in *Oryza rufipogon* and *Oryza sativa*. *Evol. Appl.* 13 2284–2299. 10.1111/eva.13022 33005224 PMC7513716

[B35] Hijmans R. J. Cameron S. E. Parra J. L. Jones P. G. Jarvis A. (2005). Very high resolution interpolated climate surfaces for global land areas. *Int. J. Climatol.* 25 1965–1978.

[B36] Ho S. Y. W. (2007). Calibrating molecular estimates of substitution rates and divergence times in birds. *J. Avian Biol.* 38 409–414.

[B37] Hoban S. Bruford M. Jackson J. D. Lopes-Fernandes M. Heuertz M. Hohenlohe P. A. (2020). Genetic diversity targets and indicators in the CBD post-2020 global biodiversity framework must be improved. *Biol. Conserv.* 248:108654.

[B38] Hou Z. G. Sket B. Li S. Q. (2014). Phylogenetic analyses of *Gammaridae crustacean* reveal different diversification patterns among sister lineages in the *Tethyan region*. *Cladistics* 30 352–365. 34794244 10.1111/cla.12055

[B39] Hu H. Hu Q. J. Al-Shehbaz I. A. Luo X. Zeng T. T. Guo X. Y. (2016). Species delimitation and interspecific relationships of the genus *Orychophragmus* (*Brassicaceae*) inferred from whole chloroplast genomes. *Frontiers in Plant Science* 7:1826. 10.3389/fpls.2016.01826 PMC5138468 27999584

[B40] Huang R. Jiang L. Zheng J. Wang T. Wang H. Huang Y. (2013). Genetic bases of rice grain shape: so many genes, so little known. *Trends Plant Sci.* 18 218–226. 10.1016/j.tplants.2012.11.001 23218902

[B41] Huang X. Kurata N. Wei X. Wang Z. X. Wang A. Zhao Q. (2012). A map of rice genome variation reveals the origin of cultivated rice. *Nature* 490 497–501. 10.1038/nature11532 23034647 PMC7518720

[B42] Katoh K. Standley D. M. (2013). MAFFT multiple sequence alignment software version 7: improvements in performance and usability. *Mol. Biol. Evolu.* 30 772–780. 10.1093/molbev/mst010 23329690 PMC3603318

[B43] Khoshbakht K. Hammer K. (2008). How many plant species are cultivated? *Gene. Res. Crop Evolu.* 55 925–928. 10.1007/s10722-008-9368-0

[B44] Kovi M. R. Zhang Y. Yu S. Yang G. Yan W. Xing Y. (2011). Candidacy of a chitin-inducible gibberellin-responsive gene for a major locus affecting plant height in rice that is closely linked to green revolution gene sd1. *Theor. Appl. Genet.* 123 705–714. 10.1007/s00122-011-1620-x 21637999

[B45] Kumar S. Stecher G. Tamura K. (2016). MEGA7: molecular evolutionary genetics analysis version 7.0 for bigger datasets. *Mol. Biol. Evolu.* 33 1870–1874. 10.1093/molbev/msw054 PMC8210823 27004904

[B46] Landis M. J. Matzke N. J. Moore B. R. Huelsenbeck J. P. (2013). Bayesian analysis of biogeography when the number of areas is large. *Syst. Biol.* 62 789–804. 23736102 10.1093/sysbio/syt040 PMC4064008

[B47] Leebens-Mack J. H. Barker M. S. Carpenter E. J. Deyholos M. K. Gitzendanner M. A. Graham S. W. (2019). One thousand plant transcriptomes and the phylogenomics of green plants. *Nature* 574 679–685. 10.1038/s41586-019-1693-2 31645766 PMC6872490

[B48] Leigh J. W. Bryant D. (2015). POPART: full-feature software for haplotype network construction. *Methods Ecol. Evolu.* 6 1110–1116.

[B49] Li C. B. Zhou A. L. Sang T. (2006). Rice domestication by reducing shattering. *Science* 311 1936–1939. 10.1126/science.1123604 16527928

[B50] Li H. Durbin R. (2010). Fast and accurate long-read alignment with burrows–wheeler transform. *Bioinformatics* 26 589–595. 20080505 10.1093/bioinformatics/btp698 PMC2828108

[B51] Li H. T. Yi T. S. Gao L. M. Ma P. F. Zhang T. Yang J. B. (2019). Origin of angiosperms and the puzzle of the Jurassic gap. *Nat. Plants* 5 461–470. 10.1038/s41477-019-0421-0 31061536

[B52] Li R. Li M. Ashraf U. Liu S. Zhang J. (2019). Exploring the relationships between yield and yield-related traits for rice varieties released in China from 1978 to 2017. *Front. Plant Sci.* 10:543. 10.3389/fpls.2019.00543 31134107 PMC6514245

[B53] Liu H. B. Shi J. P. Cai Z. X. Huang Y. M. Lv M. L. Du H. L. (2020). Evolution and domestication footprints uncovered from the genomes of coix. *Mol. Plant* 13 295–308. 10.1016/j.molp.2019.11.009 31778842

[B54] Liu L. Wang Z. Huang L. Wang T. Su Y. (2019). Chloroplast population genetics reveals low levels of genetic variation and conformation to the central-marginal hypothesis in taxus wallichiana var. mairei, an endangered conifer endemic to China. *Ecol. Evol.* 9 11944–11956. 10.1002/ece3.5703 31695899 PMC6822043

[B55] Liu X. M. Fu Y. X. (2015). Exploring population size changes using SNP frequency spectra. *Nat. Genet.* 47 555–559. 25848749 10.1038/ng.3254 PMC4414822

[B56] Londo J. P. Chiang Y. C. Hung K. H. Chiang T. Y. Schaal B. A. (2006). Phylogeography of Asian wild rice, *Oryza rufipogon*, reveals multiple independent domestications of cultivated rice, *Oryza sativa*. *Proc. Natl. Acad. Sci. U.S.A.* 103 9578–9583. 10.1073/pnas.0603152103 16766658 PMC1480449

[B57] Lu S. J. Dong L. D. Fang C. Liu S. L. Kong L. P. Cheng Q. (2020). Stepwise selection on homeologous PRR genes controlling flowering and maturity during soybean domestication. *Nat. Genet.* 52:428. 10.1038/s41588-020-0604-7 32231277

[B58] Ma X. Fan J. Wu Y. Zhao S. Zheng X. Sun C. (2020). Whole-genome de novo assemblies reveal extensive structural variations and dynamic organelle-to-nucleus DNA transfers in African and Asian rice. *Plant J.* 104 596–612. 10.1111/tpj.14946 32748498 PMC7693357

[B59] Matzke N. J. (2013). Probabilistic historical biogeography: new models for founder event speciation, imperfect detection, and fossils allow improved accuracy and model-testing. *Front. Biogeogr.* 5:242–248.

[B60] Miller A. J. Gross B. L. (2011). From forest to field: perennial fruit crop domestication. *Am. J. Bot.* 98 1389–1414. 10.3732/ajb.1000522 21865506

[B61] Molina J. Sikora M. Garud N. Flowers J. M. Rubinstein S. Reynolds A. (2011). Molecular evidence for a single evolutionary origin of domesticated rice. *Proc. Natl. Acad. Sci. U.S.A.* 108 8351–8356. 10.1073/pnas.1104686108 21536870 PMC3101000

[B62] Moner A. M. Furtado A. Henry R. J. (2020). Two divergent chloroplast genome sequence clades captured in the domesticated rice gene pool may have significance for rice production. *BMC Plant Biol.* 20:472. 10.1186/s12870-020-02689-6 33054735 PMC7558744

[B63] Nguyen L.-T. Schmidt H. A. von Haeseler A. Minh B. Q. (2014). IQ-TREE: a fast and effective stochastic algorithm for estimating maximum-likelihood phylogenies. *Mol. Biol. Evolu.* 32 268–274. 10.1093/molbev/msu300 PMC4271533 25371430

[B64] Palumbi S. R. Cipriano F. Hare M. P. (2001). Predicting nuclear gene coalescence from mitochondrial data: the three-times rule. *Evolution* 55 859–868. 11430646 10.1554/0014-3820(2001)055[0859:pngcfm]2.0.co;2

[B65] Petit N. Barbadilla A. (2009). Selection efficiency and effective population size in *Drosophila* species. *J. Evol. Biol.* 22 515–526. 10.1111/j.1420-9101.2008.01672.x 19170822

[B66] Phillips S. J. Anderson R. P. Dudik M. Schapire R. E. Blair M. E. (2017). Opening the black box: an open-source release of maxent. *Ecography* 40 887–893.

[B67] Pollmann B. Jacomet S. Schlumbaum A. (2005). Morphological and genetic studies of waterlogged *Prunus* species from the roman vicus tasgetium (Eschenz, Switzerland). *J. Archaeol. Sci.* 32 1471–1480.

[B68] Razifard H. Ramos A. Della Valle A. L. Bodary C. Goetz E. Manser E. J. (2020). Genomic evidence for complex domestication history of the cultivated tomato in Latin America. *Mol. Biol. Evol.* 37 1118–1132. 10.1093/molbev/msz297 31912142 PMC7086179

[B69] Ree R. H. Smith S. A. (2008). Maximum likelihood inference of geographic range evolution by dispersal, local extinction, and cladogenesis. *Syst. Biol.* 57 4–14. 18253896 10.1080/10635150701883881

[B70] Rojas-Barrera I. C. Wegier A. Gonzalez J. D. S. Owens G. L. Rieseberg L. H. Pinero D. (2019). Contemporary evolution of maize landraces and their wild relatives influenced by gene flow with modern maize varieties. *Proc. Natl. Acad. Sci. U.S.A.* 116 21302–21311. 10.1073/pnas.1817664116 31570572 PMC6800366

[B71] Ronquist F. (1997). Dispersal-vicariance analysis: a new approach to the quantification of historical biogeography. *Syst. Biol.* 46 195–203.

[B72] Roux F. Mary-Huard T. Barillot E. Wenes E. Botran L. Durand S. (2016). Cytonuclear interactions affect adaptive traits of the annual plant *Arabidopsis thaliana* in the field. *Proc. Natl. Acad. Sci. U.S.A.* 113 3687–3692. 10.1073/pnas.1520687113 26979961 PMC4822599

[B73] Rozas J. Ferrer-Mata A. Sánchez-DelBarrio J. C. Guirao-Rico S. Librado P. Ramos-Onsins S. E. (2017). DnaSP 6: DNA sequence polymorphism analysis of large data sets. *Mol. Biol. Evol.* 34 3299–3302. 29029172 10.1093/molbev/msx248

[B74] Sang T. Ge S. (2007). Genetics and phylogenetics of rice domestication. *Curr. Opin. Genet. Dev.* 17 533–538. 10.1016/j.gde.2007.09.005 17988855

[B75] Schlumbaum A. Tensen M. Jaenicke-Despres V. (2008). Ancient plant DNA in archaeobotany. *Vegetation History Archaeobotany* 17 233–244.

[B76] Scott M. F. Botigue L. R. Brace S. Stevens C. J. Mullin V. E. Stevenson A. (2019). A 3,000-year-old egyptian emmer wheat genome reveals dispersal and domestication history. *Nat. Plants* 5 1120–1128. 10.1038/s41477-019-0534-5 31685951 PMC6858886

[B77] Smarda P. Bures P. Horova L. Leitch I. J. Mucina L. Pacini E. (2014). Ecological and evolutionary significance of genomic GC content diversity in monocots. *Proc. Natl. Acad. Sci. U.S.A.* 111 E4096–E4102. 25225383 10.1073/pnas.1321152111 PMC4191780

[B78] Smith B. D. (1998). *The Emergence of Agriculture.* New York: Scientific American Library, A Division of HPHLP.

[B79] Smith K. F. Sax D. F. Lafferty K. D. (2006). Evidence for the role of infectious disease in species extinction and endangerment. *Conserv. Biol.* 20 1349–1357. 17002752 10.1111/j.1523-1739.2006.00524.x

[B80] Snyder C. W. (2016). Evolution of global temperature over the past two million years. *Nature* 538:226. 10.1038/nature19798 27669024

[B81] South P. F. Cavanagh A. P. Liu H. W. Ort D. R. (2019). Synthetic glycolate metabolism pathways stimulate crop growth and productivity in the field. *Science* 363:45. 10.1126/science.aat9077 PMC7745124 30606819

[B82] Sweeney M. T. Thomson M. J. Pfeil B. E. McCouch S. (2006). Caught red-handed: RC encodes a basic helix-loop-helix protein conditioning red pericarp in rice. *Plant Cell* 18 283–294. 10.1105/tpc.105.038430 16399804 PMC1356539

[B83] Takhtajan A. (1986). *Floristic Regions of the World.* California: University of California Press.

[B84] Tanaka M. Cabrera V. M. Gonzalez A. M. Larruga J. M. Takeyasu T. Fuku N. (2004). Mitochondrial genome variation in eastern Asia and the peopling of Japan. *Genome Res.* 14 1832–1850. 10.1101/gr.2286304 15466285 PMC524407

[B85] Tembrock L. R. Simmons M. P. Richards C. M. Reeves P. A. Reilley A. Curto M. A. (2017). Phylogeography of the wild and cultivated stimulant plant qat (*Catha edulis*, celastraceae) in areas of historical cultivation. *Am. J. Bot.* 104 538–549. 10.3732/ajb.1600437 28411209

[B86] Tillich M. Lehwark P. Pellizzer T. Ulbricht-Jones E. S. Fischer A. Bock R. (2017). GeSeq - versatile and accurate annotation of organelle genomes. *Nucleic Acids Res.* 45 W6–W11. 28486635 10.1093/nar/gkx391 PMC5570176

[B87] Tonti-Filippini J. Nevill P. G. Dixon K. Small I. (2017). What can we do with 1000 plastid genomes? *Plant J.* 90 808–818. 28112435 10.1111/tpj.13491

[B88] Valliyodan B. Ye H. Song L. Murphy M. Shannon J. G. Nguyen H. T. (2017). Genetic diversity and genomic strategies for improving drought and waterlogging tolerance in soybeans. *J. Exp. Bot.* 68 1835–1849. 10.1093/jxb/erw433 27927997

[B89] Vaughan D. A. Lu B. R. Tomooka N. (2008). The evolving story of rice evolution. *Plant Sci.* 174 394–408. 10.1016/j.plantsci.2008.01.016

[B90] Vikas V. K. Kumar S. Archak S. Tyagi R. K. Kumar J. Jacob S. (2020). Screening of 19,460 genotypes of wheat species for resistance to powdery mildew and identification of potential candidates using focused identification of germplasm strategy (FIGS). *Crop Sci.* 60 2857–2866. 10.1002/csc2.20196

[B91] Wagner S. Lagane F. Seguin-Orlando A. Schubert M. Leroy T. Guichoux E. (2018). High-throughput DNA sequencing of ancient wood. *Mol. Ecol.* 27 1138–1154. 29412519 10.1111/mec.14514 PMC5896730

[B92] Wang H. Takano T. Liu S. K. (2018). Screening and evaluation of saline-alkaline tolerant germplasm of rice (*Oryza sativa* L.) in soda saline-alkali soil. *Agron. Basel* 8:205. 10.3390/agronomy8100205

[B93] Wang W. S. Mauleon R. Hu Z. Q. Chebotarov D. Tai S. S. Wu Z. C. (2018). Genomic variation in 3,010 diverse accessions of Asian cultivated rice. *Nature* 557:43. 10.1038/s41586-018-0063-9 29695866 PMC6784863

[B94] Wang K. Li M. Y. Hakonarson H. (2010). ANNOVAR: functional annotation of genetic variants from high-throughput sequencing data. *Nucleic Acids Res.* 38:e164. 10.1093/nar/gkq603 PMC2938201 20601685

[B95] Wick R. R. Schultz M. B. Zobel J. Holt K. E. (2015). Bandage: interactive visualization of de novo genome assemblies. *Bioinformatics* 31 3350–3352. 10.1093/bioinformatics/btv383 26099265 PMC4595904

[B96] Wilkes G. Williams J. T. (1983). Current status of crop plant germplasm. *Crit. Rev. Plant Sci.* 1 133–181. 10.1080/07352688309382175

[B97] Wu Z. Y. Wu S. G. (1996). “A proposal for a new floristic kingdom (realm) — the E. Asiatic kingdom, its delimitation and characteristics,” in *Floristic Characteristics and Diversity of East Asian Plants*, eds Zhang A. L. Wu S. G. (Beijing, Berlin: China Higher Education Press, Springer-Verlag), 3–42.

[B98] Yu Y. Harris A. J. Blair C. He X. J. (2015). RASP (reconstruct ancestral state in phylogenies): a tool for historical biogeography. *Mol. Phylogenet. Evol.* 87 46–49. 25819445 10.1016/j.ympev.2015.03.008

[B99] Yu Y. Harris A. J. He X. J. (2010). S-DIVA (statistical dispersal-vicariance analysis): a tool for inferring biogeographic histories. *Mol. Phylogenet. Evol.* 56 848–850. 20399277 10.1016/j.ympev.2010.04.011

