# Supplementary material for: The History and Diversity of Rice Domestication as Resolved From 1464 Complete Plastid Genomes
Source: Front Plant Sci. 2021 Nov 18;12:781793. doi: 10.3389/fpls.2021.781793 (PMC8637288; doi:10.3389/fpls.2021.781793)
Supplement: Supplementary file 2 [file Table_2.docx]

**Supplemental tables**

**Table S2 Published plastomes of *Oryza* species used in this study**

| ID | type | Group | Origin | Lat | Long | Plastomic  haplotype | Used in time tree construction |
| --- | --- | --- | --- | --- | --- | --- | --- |
| JN005831 | OM | O-I | India | NA | NA | Hap_245 | Yes |
| JN005832 | OR | O-unc | Vietnam | 9.9896 | 105.66472 | Hap_119 | No |
| JN005833 | OR | O-I | Australia | -18.2 | 142.87 | Hap_257 | No |
| KF359906 | OM | O-I | India | NA | NA | Hap_248 | Yes |
| KF359907 | OL | O-II | NA | NA | NA | Hap_264 | Yes |
| KF428978 | OR | O-I | Australia | NA | NA | Hap_255 | No |
| KF562709 | OR | Or-w-I | China | NA | NA | Hap_112 | No |
| KM088024 | OL | O-II | NA | NA | NA | Hap_262 | No |
| KM103373 | OM | O-I | India | NA | NA | Hap_251 | No |
| KM103374 | Oglu | O-II | NA | NA | NA | Hap_258 | No |
| KM881639 | Ogla | Or-wj-II | NA | NA | NA | Hap_32 | Yes |
| KM881640 | Oglu | O-II | NA | NA | NA | Hap_259 | No |
| KM881641 | OL | O-II | China | NA | NA | Hap_261 | No |
| KM881642 | OL | O-II | India | NA | NA | Hap_263 | Yes |
| KR364803 | Oglu | O-II | NA | NA | NA | Hap_260 | Yes |
| NC_027676 | OP | Op | Thailand | NA | NA | Hap_265 | Yes |
| MZ151337 | Ogla | O-unc | WARDA | NA | NA | Hap_239 | No |
| OK662594 | Ind | Or-w-II | China | NA | NA | Hap_168 | Yes |
| X15901 | Jap | O-unc | Japan | NA | NA | Hap_266 | No |

Note: WARDA, African rice center.

**Table S4 Functional variations in the CDS of protein coding genes**

| Type of variations | Frequency |
| --- | --- |
| Frameshift deletions | 3 |
| Frameshift insertions | 2 |
| Frameshift substitutions | 1 |
| Non-frameshift deletions | 2 |
| Non-frameshift insertions | 3 |
| Non-frameshift block substitutions | 10 |
| Nonsynonymous SNVs | 113 |
| Synonymous SNVs | 215 |
| Stop-codon-gain variations | 2 |
|  |  |
| Total | 351 |

| Genetic  cluster | *japonica* | *indica* | *aromatic* | *aus* | Intermediate | Common wild rice | Other |
| --- | --- | --- | --- | --- | --- | --- | --- |
| Or-wj-I | 250 (0.817/0.522) | 27 (0.088/0.058) | 13 (0.042/0.194) | 6 (0.02/0.058) | 8 (0.026/0.348) | 2 (0.007/0.007) | 0 (0/0) |
| Or-wj-II | 172 (0.619/0.359) | 73 (0.263/0.157) | 6 (0.022/0.09) | 10 (0.036/0.096) | 5 (0.018/0.217) | 11 (0.04/0.037) | 1 (0.004/0.036) |
| Or-wj-III | 13 (0.072/0.027) | 37 (0.206/0.08) | 39 (0.217/0.582) | 19 (0.106/0.183) | 3 (0.017/0.13) | 69 (0.383/0.231) | 0 (0/0) |
| Or-w-I | 0 (0/0) | 0 (0/0) | 0 (0/0) | 0 (0/0) | 0 (0/0) | 34 (1/0.114) | 0 (0/0) |
| Or-wi-I | 39 (0.096/0.081) | 280 (0.691/0.603) | 8 (0.02/0.119) | 61 (0.151/0.587) | 6 (0.015/0.261) | 11 (0.027/0.037) | 0 (0/0) |
| Or-wi-II | 2 (0.028/0.004) | 31 (0.437/0.067) | 0 (0/0) | 5 (0.07/0.048) | 1 (0.014/0.043) | 32 (0.451/0.107) | 0 (0/0) |
| Or-w-II | 2 (0.017/0.004) | 11 (0.092/0.024) | 1 (0.008/0.015) | 2 (0.017/0.019) | 0 (0/0) | 103 (0.866/0.344) | 0 (0/0) |
| Or-w-III | 0 (0/0) | 3 (0.25/0.006) | 0 (0/0) | 0 (0/0) | 0 (0/0) | 9 (0.75/0.03) | 0 (0/0) |
| Or-w-IV | 0 (0/0) | 1 (0.077/0.002) | 0 (0/0) | 1 (0.077/0.01) | 0 (0/0) | 11 (0.846/0.037) | 0 (0/0) |
| O-mixed | 1 (0.05/0.002) | 1 (0.05/0.002) | 0 (0/0) | 0 (0/0) | 0 (0/0) | 10 (0.5/0.033) | 8 (0.4/0.286) |
| O-I | 0 (0/0) | 0 (0/0) | 0 (0/0) | 0 (0/0) | 0 (0/0) | 7 (0.389/0.023) | 11 (0.611/0.393) |
| O-II | 0 (0/0) | 0 (0/0) | 0 (0/0) | 0 (0/0) | 0 (0/0) | 0 (0/0) | 7 (1/0.25) |
| Op | 0 (0/0) | 0 (0/0) | 0 (0/0) | 0 (0/0) | 0 (0/0) | 0 (0/0) | 1 (1/0.036) |

**Table S5 Number and Frequencies of different types of *Oryza* accessions detected in different genetic clusters.**

Note: Numbers outside the brackets are the numbers of accessions detected in the corresponding genetic clusters; First number in the brackets is the rate of detected accessions out of the total number of all accessions in that cluster, while the second one is the rate of detected accessions out of the total number of all accessions in that material type.

**Table S6 Model test results in the analysis of ancestral range distribution**

| Model | LnL | Num of params | *d* | *e* | *j* | AIC | △AIC | AICc | △AICc |
| --- | --- | --- | --- | --- | --- | --- | --- | --- | --- |
| DEC | -142.88 | 2.00 | 0.26 | 0.36 | 0.00 | 289.76 | 10.31 | 290.07 | 9.98 |
| DEC+J | -136.73 | 3.00 | 0.17 | 0.18 | 0.06 | 279.46 | 0.00 | 280.09 | 0.00 |
| DIVALIKE | -149.75 | 2.00 | 0.33 | 0.48 | 0.00 | 303.50 | 24.05 | 303.81 | 23.72 |
| DIVALIKE+J | -144.87 | 3.00 | 0.21 | 0.25 | 0.05 | 295.74 | 16.28 | 296.37 | 16.28 |
| BAYAREALIKE | -155.51 | 2.00 | 0.23 | 1.46 | 0.00 | 315.01 | 35.56 | 315.32 | 35.23 |
| BAYAREALIKE+J | -144.99 | 3.00 | 0.14 | 0.50 | 0.07 | 295.99 | 16.53 | 296.62 | 16.53 |

**Table S7 The Neutrality tests of plastomic variations in different genetic clusters**

| Groups | Tajima's D | *p* | Fu and Li's F test with an outgroup | *p* |
| --- | --- | --- | --- | --- |
| 00Or-wj-I | -2.14172 | < 0.01 ** | -4.92413 | <0.02 * |
| 01Or-wj-II | -2.43634 | < 0.01 ** | -4.31345 | <0.02 * |
| 02Or-wj-III | -2.47371 | < 0.001 *** | -5.46968 | <0.02 * |
| 04Or-w-I | -0.12327 | > 0.10 | -1.51455 | >0.10 |
| 05Or-wi-I | -2.48622 | < 0.001 *** | -6.69556 | <0.02 * |
| 06Or-wi-II | -1.8365 | < 0.05 * | -3.4064 | <0.02 * |
| 07Or-w-II | -1.51418 | > 0.10 | -3.02731 | <0.02 * |
| 08Or-w-III | -1.59886 | > 0.05 | -1.36715 | >0.10 |
| 09Or-w-IV | -0.42534 | > 0.10 | -0.00526 | >0.10 |
| 11O-I | -1.38465 | > 0.10 | -2.17498 | >0.05 |
| 12O-II | -0.82924 | > 0.10 | -1.70441 | >0.10 |

**Table S8 Genetic diversity within each genetic cluster of wild and cultivated rice**

| GroupID | No. of haplotypes | *S* | *Hd* | *Theta* | *k* |
| --- | --- | --- | --- | --- | --- |
| Or-wj-I | 31 | 50 | 0.744 | 0.00006 | 2.022 |
| Or-wj-II | 33 | 76 | 0.887 | 0.00009 | 4.212 |
| Or-wj-III | 38 | 96 | 0.801 | 0.00012 | 3.982 |
| Or-w-I | 16 | 83 | 0.970 | 0.00015 | 21.433 |
| Or-wi-I | 36 | 57 | 0.782 | 0.00006 | 2.454 |
| Or-wi-II | 11 | 52 | 0.866 | 0.00008 | 6.033 |
| Or-w-II | 50 | 153 | 0.987 | 0.00021 | 17.948 |
| Or-w-III | 6 | 36 | 0.803 | 0.00009 | 9.515 |
| Or-w-IV | 9 | 48 | 0.962 | 0.00012 | 14.41 |

Note: *S*, number of polymorphic (segregating) sites; *Hd*, haplotype diversity; *Theta*, Theta (per site) from *S*; *k*, number of polymorphic (segregating) sites.

**Supplemental figure legends**

**Fig. S1 Geographic distribution of the wild and cultivated rice accessions used in this study.** The sizes of green circles are approximately proportional to the number of samples in that location.

**Fig S2. Nucleotide variations in plastomes of all the rice accessions in Oryza species with AA genome.** The SNVs, Indels and structural variations (SVs) were presented on the inner tracks. The SVs consisted of fragment substitutions and length polymorphism, and large Indels longer than 50 bp.

**Fig. S3 The cross-validation (CV) errors of multiple Admixture runs with different K values for plastomes of all the 1464 accessions.** The CV error reached to the first lowest value at K=12. However, the lowest CV errors were detected at K=17 if we excluded the results with high variation between replications.

**Fig. S4 Average nucleotide diversity across plastomes of different genetic clusters, at a sliding window of 600 bp and step size of 200 bp.**

**Fig. S5 Principal component analysis (PCA, A) and multidimensional scaling (MDS, B) plot for the *Oryza* accessions.**

**Fig S6. Chronogram of the selected 61 haplotypes.** The scale is in mya.

**Fig S7. Geographic distribution among *Oryza* haplotypes used in this study**

**Fig. S8 The ancestral geographic distributions of selected common haplotypes.** The two sub-figures presented the ancestral geographic distributions as inferred by BioGeoBEARS from different prospects: the left one showed the inferred distribution of each node with highest probability while the right one showed the probabilities of all possible distributions for each node. The capital letters denoted the different geographic regions: A, East Asia; B, South Asia; C, Southeast Asia; D, Southeast Asia - islands; E, Africa; F, America.

**Fig. S9 Mismatch distribution calculated for each genetic cluster.** The plots indicate the expected and observed frequencies of pairwise differences between haplotypes within each genetic cluster.

**Figure S10 Different classifications for the wild and cultivated rice accessions based on genomic and plastomic variations.** The nuclear type of wild rice based on genomic variations were defined as described in Huang et al. (2012). The material type and genetic clusters were defined the same as in Fig. 1.

**Supplemental figures**


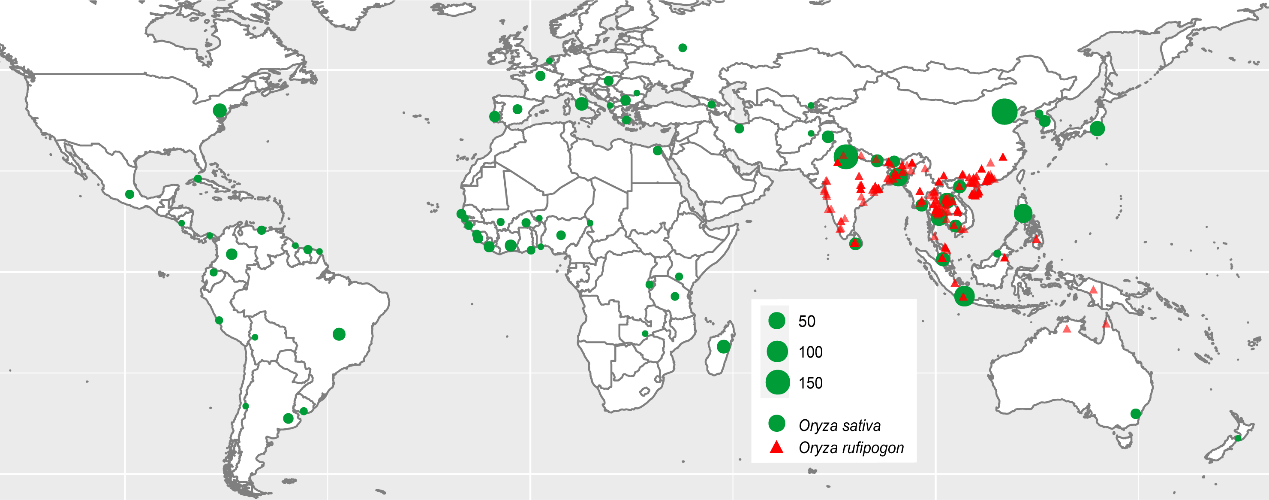


**Fig. S1 Geographic distribution of the wild and cultivated rice accessions used in this study.**


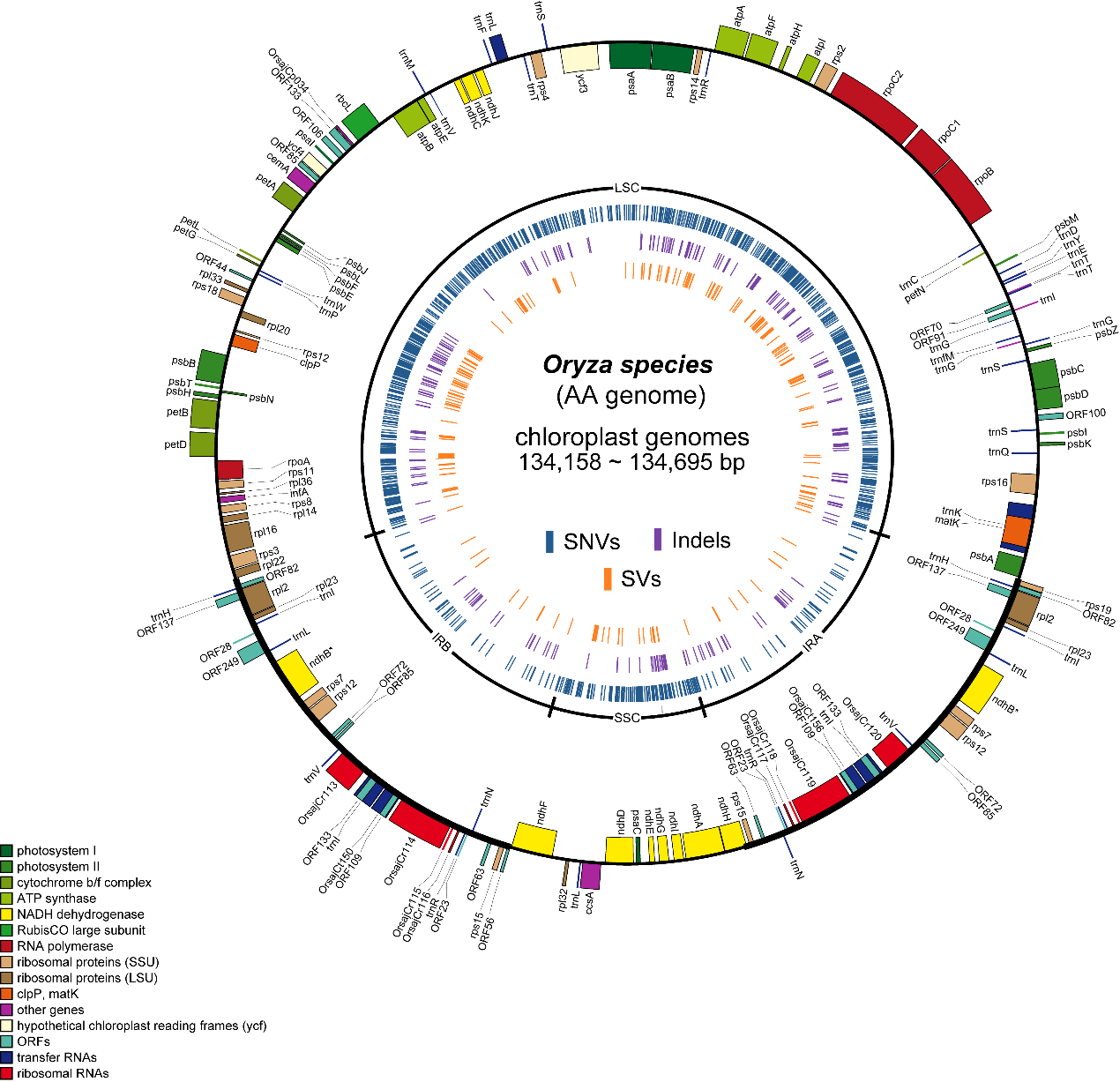


**Fig S2. Nucleotide variations in plastomes of all the rice accessions in Oryza species with AA genome.**


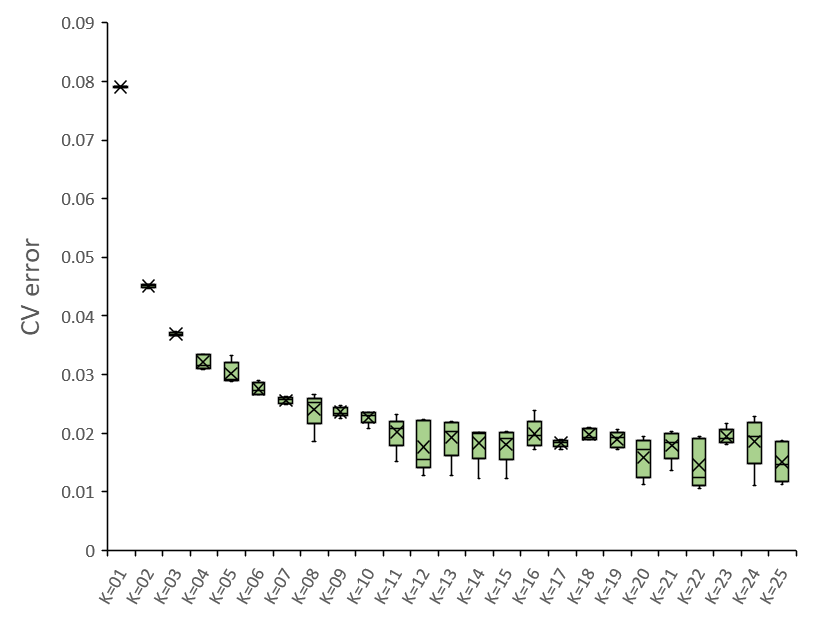


**Fig. S3 The cross-validation (CV) errors of multiple Admixture runs with different K values for plastomes of all the 1464 accessions.**


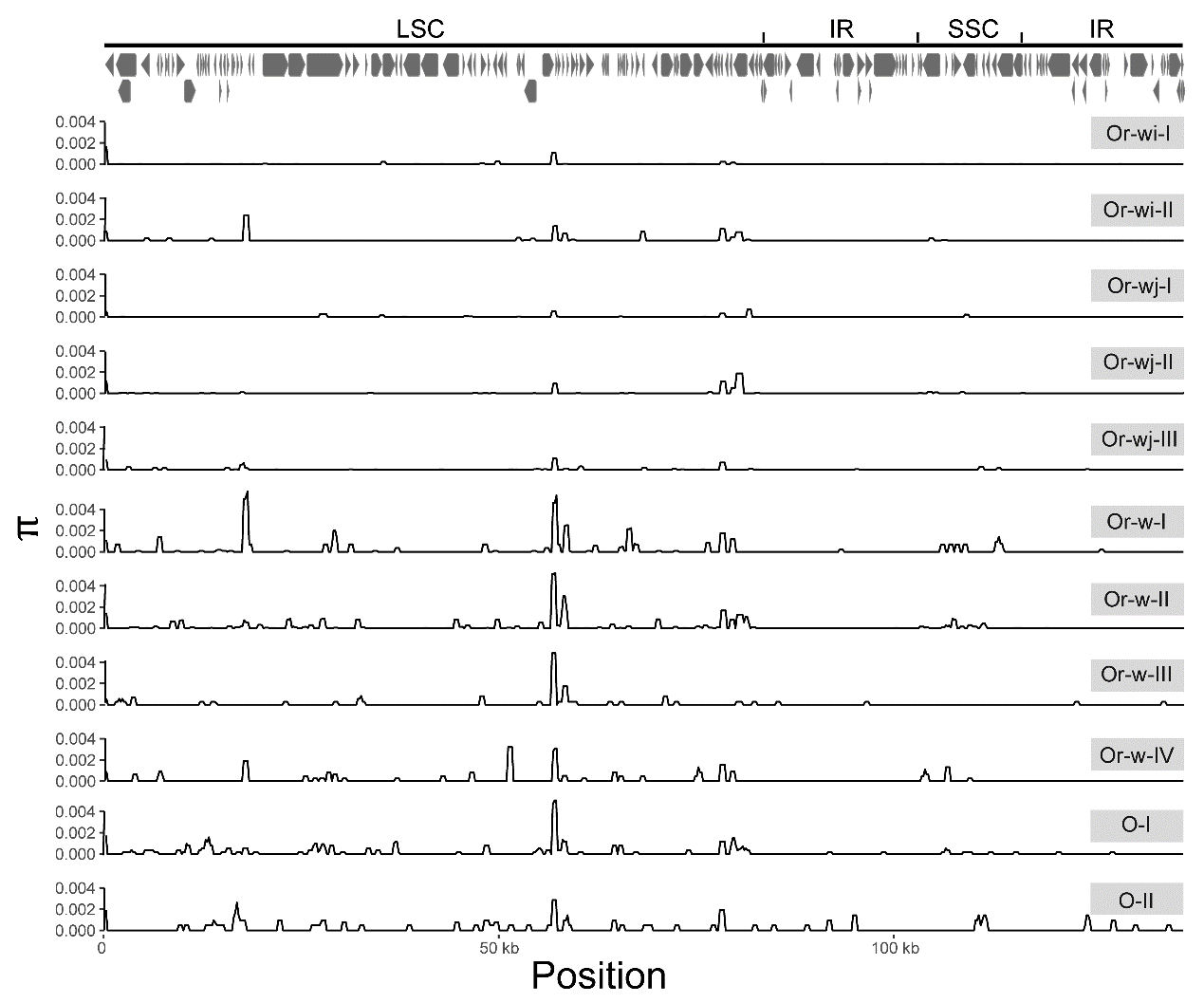


**Fig. S4 Average nucleotide diversity across plastomes from different genetic clusters using a sliding window of 600 bp and a step size of 200 bp.**


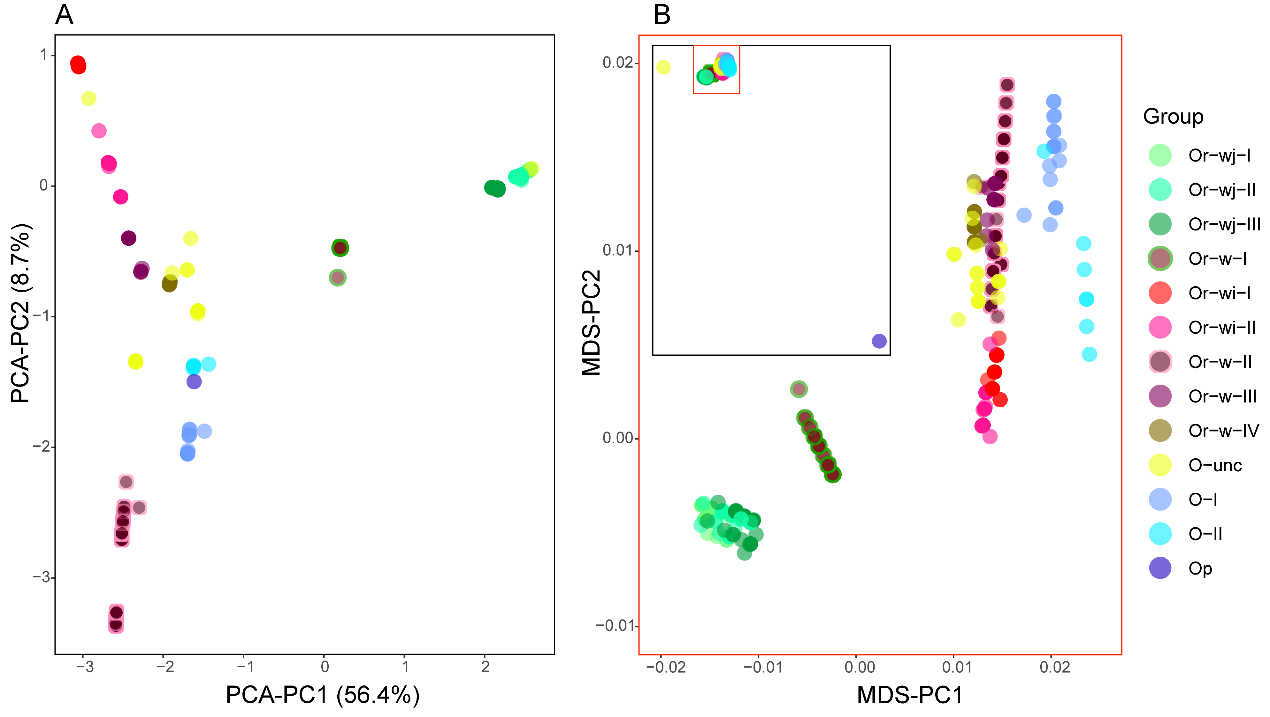


**Fig. S5 Principal component analysis (PCA, A) and multidimensional scaling (MDS, B) plot for the *Oryza* accessions**.


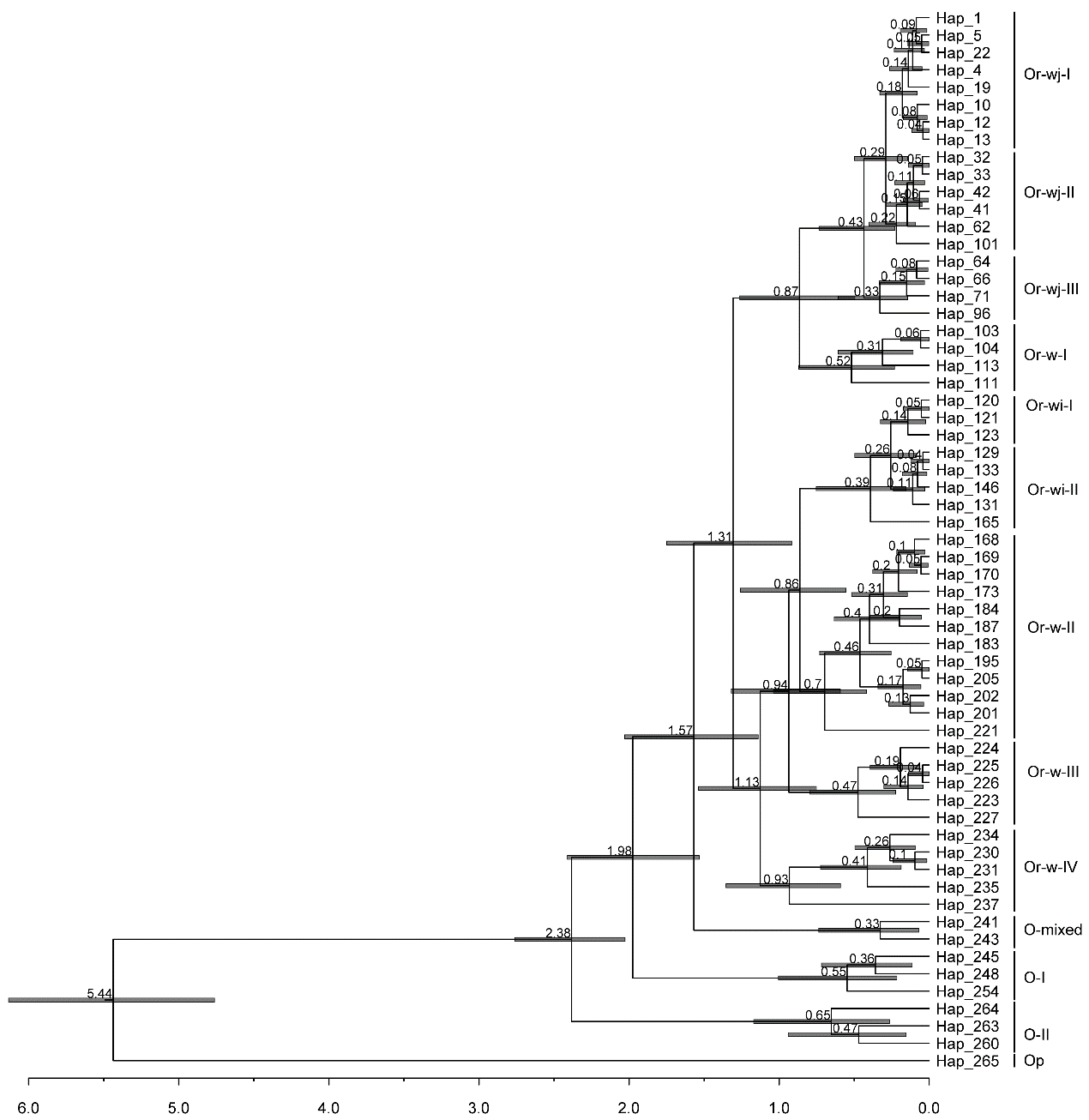


**Fig S6. Chronogram of the selected 61 haplotypes, scale is in mya**


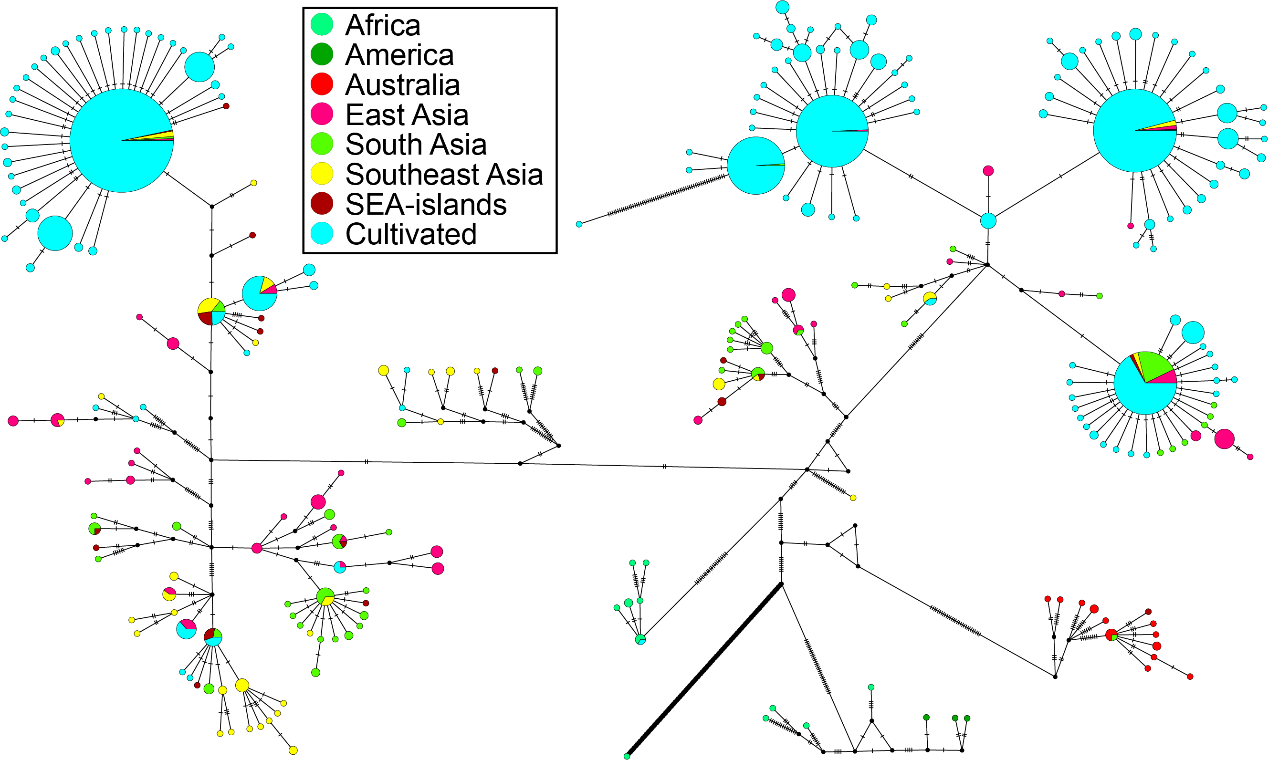


**Fig S7. Geographic distribution among *Oryza* haplotypes used in this study**


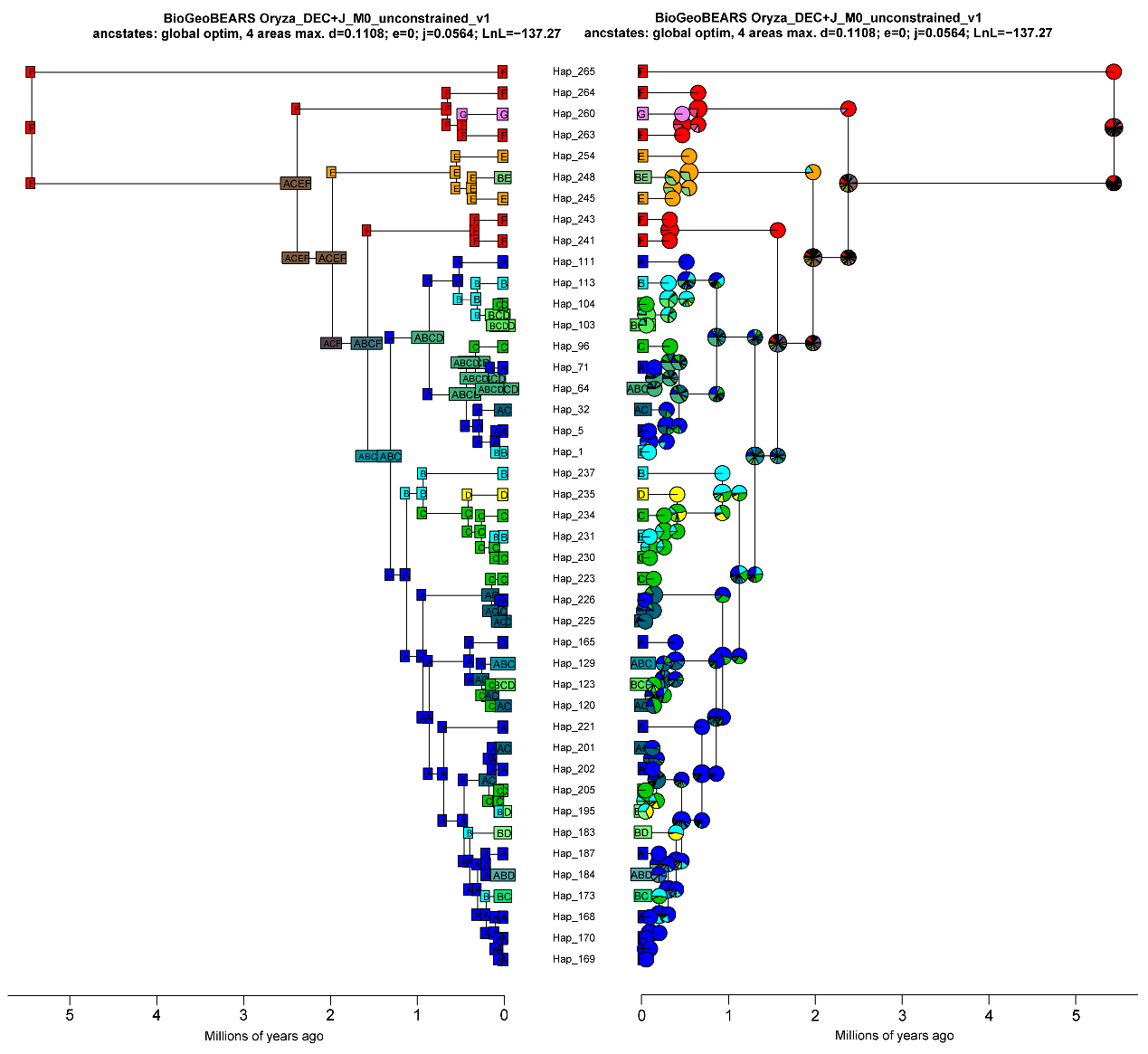


**Fig. S8 The ancestral geographic distributions of selected common haplotypes.**


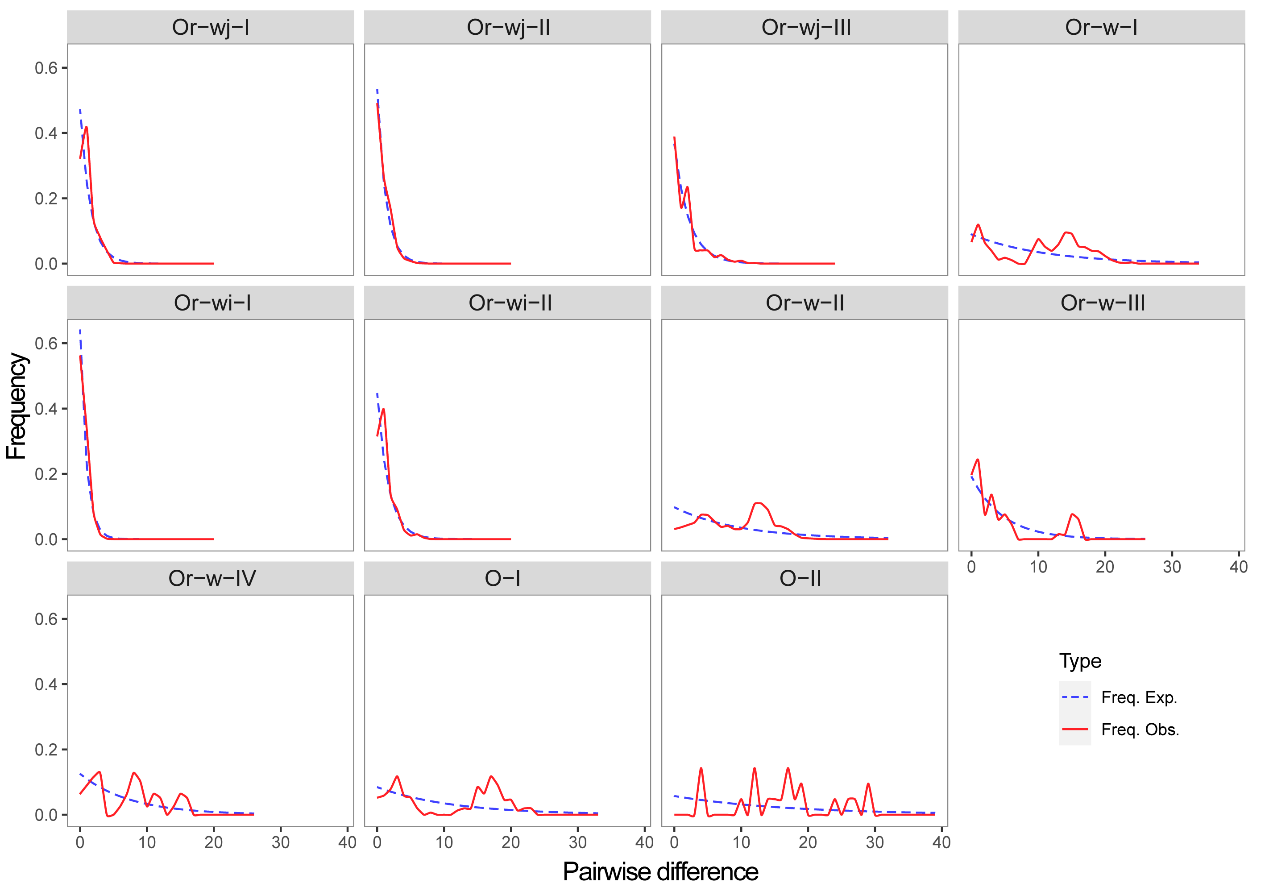


**Fig. S9 Mismatch distribution calculated for each genetic cluster.**


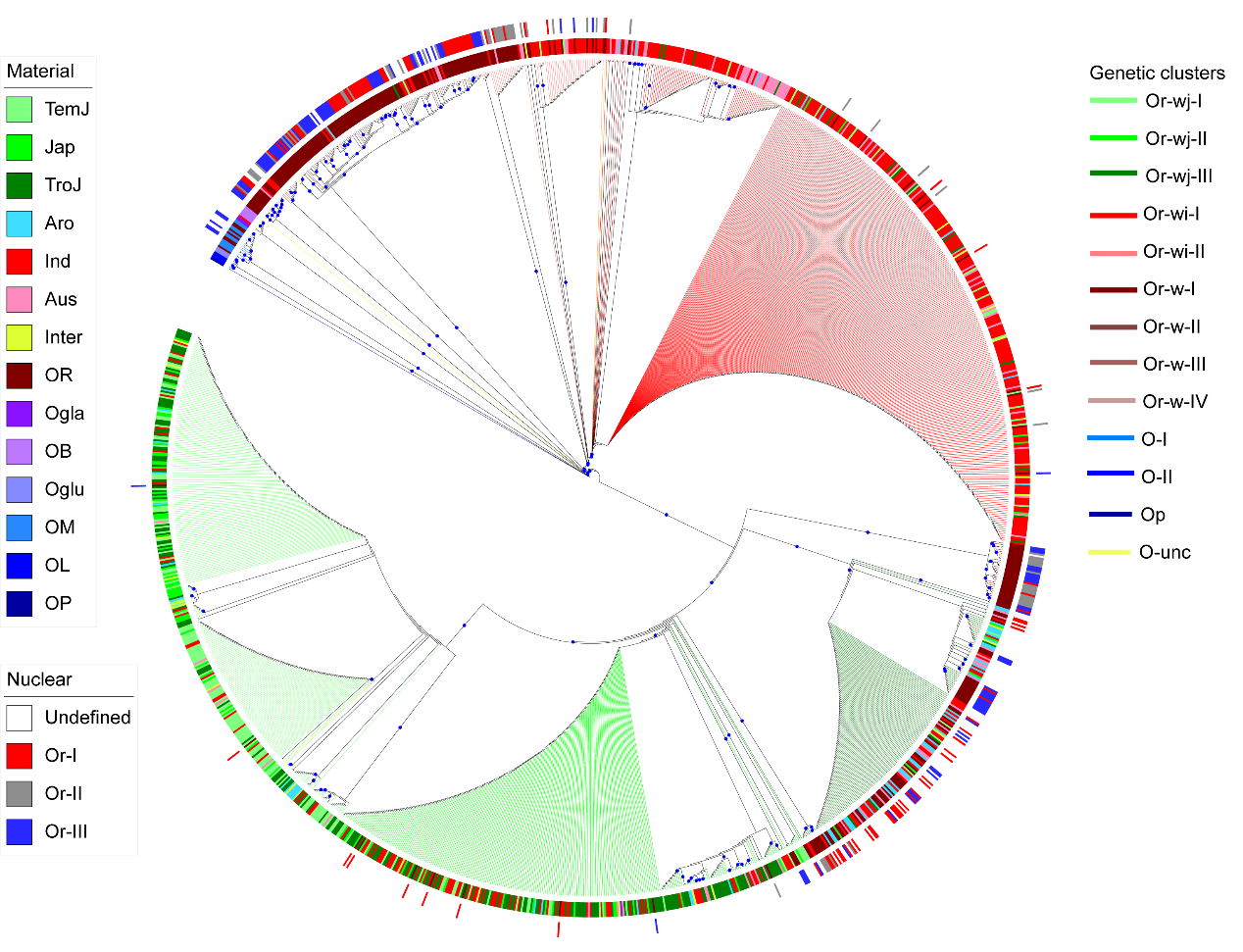


**Figure S10 Different classifications for the wild and cultivated rice accessions based on nuclear genomic and plastomic variations.**
